# Salidroside inhibits glioma cell migration and proliferation through CYP2E1-neuroglobin-cytochrome c mediated redox axis

**DOI:** 10.3389/fonc.2026.1929709

**Published:** 2026-10-01

**Authors:** Lakshmi Priya Panda, Kalpana Barhwal, Amit Kumar, Suryanarayan Biswal, Dev Kumar Tripathy, Mukund Namdev Sable, Rabi Narayan Sahu, Gaurav Chhabra

**Affiliations:** 1 Department of Physiology, All India Institute of Medical Sciences, Bhubaneswar, India; 2 Department of Human Genetics and Molecular Medicine, Central University of Punjab, Bhatinda, India; 3 Department of Pathology, All India Institute of Medical Sciences, Bhubaneswar, India; 4 Department of Neurosurgery, All India Institute of Medical Sciences, Bhubaneswar, India

**Keywords:** glioblastoma, neuroglobin, oxidative stress, redox imbalance, salidroside

## Abstract

**Background:**

Glioblastoma (GB) represents one of the most aggressive primary brain tumors characterized by poor prognosis, high relapse and resistance to conventional therapies. Salidroside, a naturally occurring glucoside derived from Rhodiola rosea, has been reported to exhibit pro-apoptotic and anti-proliferative properties across multiple cancer models; however, its mechanistic basis in glioma and its therapeutic potential remain incompletely characterized.

**Methods:**

Neuroglobin (Ngb) protein expression was analyzed in 101 glioma brain tissue specimens and 5 normal brain tissues by western blot and immunohistochemical staining. In silico analyses of oxidized and reduced state of Ngb with salidroside was performed along with pharmacokinetics and toxicity predictions to computationally evaluate possible binding interactions and drug-like properties. Further, to determine the underlying mechanisms, *in vitro* assays were performed in U87MG and LN229 human glioblastoma cells treated with salidroside. MTT assay, transwell assay, Annexin V/PI staining, mitochondrial membrane potential (MMP), CYP2E1 activity, intracellular NADPH levels, Fe2+/Fe3+ ratio and markers of oxidative stress including ROS assay and lipid peroxidation were also measured. The mechanism of Ngb aided apoptosis was investigated by protein expression of Ngb, caspase 3 and cytosolic cytochrome c in glioma cells.

**Results:**

Ngb expression in glioma tissues samples significantly downregulated with increasing tumour grade as determined by immunoreactivity and western blot analysis. In-silico analyses predicted a possible interaction of salidroside with reduced state of Ngb, *In vitro* studies suggested significant reduction in cell viability, migration and invasion in both U87MG and LN229 cells on supplementation with salidroside. These effects were accompanied by increased CYP2E1 activity, altered intracellular NADPH levels indicating induction of oxidative stress. Mitochondrial dysfunction, manifested as reduced MMP, elevated lipid peroxidation and increased cytosolic cytochrome c release indicated activation of intrinsic apoptotic pathway. Computational analyses additionally predicted a preferential interaction with the reduced form of Ngb, providing a mechanistic hypothesis warranting experimental validation.

**Conclusion:**

Salidroside exerts multi-faceted anti-cancer effects in glioblastoma cells, potentially involving upregulation of Ngb levels in glioma cells, modulation of redox homeostasis, mitochondrial integrity and intrinsic apoptotic signaling.

## 1 Introduction

Gliomas constitute the most common primary brain tumors in adults, of which glioblastoma (GB) represents the most prevalent and biologically aggressive subtype. According to the Central Brain Tumor Registry of the United States (CBTRUS) Statistical Report on Primary Brain and Other Central Nervous System (CNS) tumors, GB carries an incidence of 3.2 per 100,000 population, underscoring its considerable public health burden (1). Within the Indian subcontinent, the incidence of CNS tumors is reported to range between 5 to 10 per 100,000 population, with epidemiological surveillance data indicating a discernible upward trajectory in occurrence over recent trends. Collectively, these neoplasms account for approximately 2% of all malignancies (2). The prognosis associated with GB remains grave, with a median survival of 15-23 months from the time of diagnosis to death (3). This unfavorable clinical trajectory is attributable to a constellation of pathological features intrinsic to GB, including extensive vascular proliferation, focal necrosis, chemoresistance, intratumoral heterogeneity, and infiltrative pattern which makes it formidable to conventional therapeutic regimens (4). This bleak outcome has fostered focused research towards revealing novel insights into the hypoxic microenvironment of GB in order to facilitate the development of new treatment strategies.

Despite our growing understanding of the underlying mechanisms pertaining to GB, the current standard of care comprising surgical resection followed by concurrent chemoradiotherapy, has remained largely unchanged over the past two decades. This therapeutic stagnation underscores an urgent and unmet clinical need to identify and develop novel therapeutic strategies, capable of circumventing the refractory hindrances to improve GB survival rate.

In recent years, plant-derived pharmaceuticals have garnered considerable scientific interest as adjunctive anti-cancer agents demonstrating therapeutic potential across multiple malignancy models. The pharmacological activity of medicinal plants is largely attributed to diverse repertoire of bioactive compounds, which could modulate immune-mediate anti-cancer mechanisms by suppressing angiogenesis, neovascularization and metastatic cascade activation. Amongst the trans-himalayan herbs, salidroside, which is the bioactive ingredient found in the roots of Rhodiola sp. has been documented to protect against hypoxic/ischemic injury by increasing cell survival and angiogenesis while reducing oxidative stress and inflammation (5). Our previous studies have suggested salidroside-mediated mitophagy and its potential as a therapeutic and prophylactic molecule for hypoxia-induced neurodegeneration (6). On the other hand, few studies have reported that salidroside inhibits proliferation and induces G0/G1 cell cycle arrest in glioma U251 and U87MG cells (7, 8). However, the therapeutic potential and the underlying anti-glioma mechanism of salidroside in ameliorating stemness, proliferation, invasion and metastasis of GB remain to be explored.

As a consequence of its inherent aggressive, invasive and proliferative behavior, GB, can remodel its surrounding microenvironment, through coordinated interactions between cellular and non-cellular components fostering chemoresistance, cell proliferation, migration, and invasion (9). Though the cellular components of the tumor microenvironment viz. glioblastoma stem-like cells, endothelial cells, microglia, astrocytes, and neurons contribute towards the infiltration and invasion characteristics of GB, the non-cellular component, hypoxia in particular, has emerged as a principal orchestrator of tumor malignancy. The tumor cells of GB have the ability to adapt and endure in the hypoxic niche by triggering putative mediators that could alter the cellular redox status promoting tumor aggressiveness and shifting cellular metabolism from oxidative phosphorylation to glycolysis (10). In this context, Neuroglobin (Ngb), a monomeric protein widely expressed in the CNS has emerged as an important mediator in hypoxia-mediated cellular signaling. In a recent finding, it was reported that Ngb is downregulated in GB, and that its downregulation was positively correlated with poor patient survival (11).

In lieu of the above, the aim of the present study is to explore the mechanisms underlying anti-glioma effect of salidroside and the regulatory role of Ngb in salidroside mediated anti-cancerous effect. We, hypothesize that salidroside alters the redox status of Ngb and mitochondrial bioenergetics in U87MG and LN229 glioma cells adjuring them to undergo apoptosis. The study thus investigates a novel perspective on the therapeutic potential of salidroside in inhibiting cell migration, invasion and attenuating the metabolic energy requirements critical to tumor survival, thereby collectively offering a biologically grounded rationale for its further evaluation as an adjunctive therapeutic strategy in GB management.

## 2 Materials and methods

### 2.1 Patients and tissue sample collection

All experimental protocols were reviewed and approved by the Institutional Ethics Committee (IEC) of All India Institute of Medical Sciences (AIIMS); Bhubaneswar vide reference number IEC/AIIMSBBSR/PhDThesis/2023/03. A total of 101 adult glioma (WHO grade 2 astrocytoma and oligodendroglioma -26, WHO grade 3 astrocytoma and oligodendroglioma -24, WHO grade 4 glioblastoma-51) patients who underwent surgical excision of the tumor and diagnosed through pathological examinations were enrolled in the study from the Department of Neurosurgery and Department of Trauma and Emergency, AIIMS, Bhubaneswar. The clinical characteristics of all the patients was obtained from medical records department (MRD), AIIMS, Bhubaneswar. The classification was done in accordance with the 2021 WHO Classification of Tumors of the Central Nervous System (WHO CNS5). The WHO grade of glioma tumors was assessed by two neuropathologists through immunohistochemical analysis of IDH-1, ATRX, p53 and MIB1. The clinico-pathological characteristics of the patients are summarized in **Table 1**. The inclusion and exclusion criteria are described below:

**Table 1:** Clinicopathologic characteristics of the patient samples.

Characteristic	Category	n
**Age**	≤70 years	96
	≥70 years	5
**Type of Tumor**	Grade II	26
	Grade III	24
	Glioblastoma	51
**IDH status**	Mutant	50
	Wild type	51
**ATRX status**	Mutant	19
	Wild type	82
**P53 status**	Wild type	46
	Mutant	55
**MIB-1 score**	1%-20%	64
	21%-40%	26
	41%-60%	7
	61%-80%	4
**Vital status**	Alive	68
	Dead	33

Inclusion Criteria

1.Histopathological and radiologically confirmed diffuse glioma aligned with WHO CNS5 (2021) classification
Age 18–75 years at the time of diagnosis/sample collection
Availability of adequate biopsy/resected tumor tissue

Exclusion Criteria

1. Paediatric-type diffuse glioma
2. Non-glial primary tumors (eg. Meningiomas, primary CNS lymphomas) or metastatic brain lesions
3. Severe head injury (recent or historical, per treating clinician’s judgment) that may confound imaging, clinical, or histopathological findings
4. Neurodegenerative disorders (e.g., Alzheimer’s disease, Parkinson’s disease, motor neuron disease) or CNS infections (active or recent)

5 cases of autopsied non-tumor adult brain specimens without any histological evidence of confounding pathology served as controls. The limited number of controls reflects the stringent inclusion criteria viz. short-post mortem interval for molecular integrity and intact structural architecture for comparison. Other confounding pathology, for instance prolonged hypoxia, infection, drug overdose etc. were also excluded. Thus, to avoid introducing unrelated pathological changes that could confound the control group molecular baseline, we excluded a substantial number of otherwise available autopsy brains.

Written informed consent was obtained from all participants prior to sample collection. None of the patients received any treatments viz. chemotherapy and/or radiotherapy before tissue collection. All tissue specimens were preserved either in RIPA buffer for immunoblotting analysis or fixed in 10% formalin for immunohistochemical (IHC) examination of desired proteins until further use.

### 2.2 In-silico analysis

#### 2.2.1 Protein retrieval, preparation, and redox-state modelling of neuroglobin

The three-dimensional crystal structure of human neuroglobin (NGB; PDB ID: 1OJ6) was downloaded from the RCSB Protein Data Bank (12) and processed using AutoDock Tools (ADT) (13). Preparation involved stripping crystallographic water molecules, adding polar hydrogens, and assigning Gasteiger partial charges (14) as standard steps to make the structure docking-ready.

Since Ngb exerts its biological effects in two distinct oxidation states (15), we generated separate receptor models for the ferrous (Fe²^+^) and ferric (Fe³^+^) forms by directly editing the heme iron parameters in the.pdbqt file. This allowed us to ask a specific question: does salidroside bind differently depending on whether the Ngb iron is in the reduced or oxidized state? Before docking, both conformations were energy-minimized in UCSF Chimera (16) using AMBER force field parameters (17) to remove any steric strain introduced during preparation.

#### 2.2.2 Ligand preparation

The three-dimensional structure of salidroside (p-hydroxyphenethyl-β-D-glucoside; PubChem CID: 159278) was obtained from PubChem (18). Using AutoDock tools (13) all hydrogens were added, gasteiger charges (14) assigned, rotatable bonds identified, and the structure exported in. pdbqt format for docking.

#### 2.2.3 Molecular docking

Docking was carried out with AutoDock 4, using the Lamarckian Genetic Algorithm (LGA) (19) with 100 independent runs and up to 2000 output conformations per run. The docking grid was centered on the heme-binding cavity, specifically encompassing the two histidine residues His64 and His96 that coordinate the heme iron in Ngb (15, 20). Docked poses were ranked by binding free energy (ΔG, kcal/mol), inhibition constant (K_i_), hydrogen bond count, and ligand efficiency.

#### 2.2.4 Visualization and interaction analysis

The top-scoring docked conformations for both redox states were analyzed using three platforms: Discovery Studio Visualizer for three-dimensional interaction mapping and hydrophobic contact visualization; LigPlot^+^ for two-dimensional hydrogen bond and hydrophobic contact diagrams; and PDBSum (21) for structural interaction profiling.

#### 2.2.5 ADMET prediction

To assess whether salidroside has realistic drug-like properties, we predicted its absorption, distribution, metabolism, excretion, and toxicity (ADMET) profile using admetSAR 2.0 (22), SwissADME (23), and pkCSM (24). Parameters of particular interest for a GB candidate included blood-brain barrier (BBB) penetration, P-glycoprotein (P-gp) substrate status, cytochrome P450 (CYP450) inhibition, and oral bioavailability. Drug-likeness was assessed using Lipinski (25), Veber (26), Egan (27), and Muegge (28) criteria.

#### 2.2.6 Toxicological assessment

Acute and organ-level toxicity was evaluated computationally using ProTox-3 (29) and pkCSM (24). Predicted LD_50_ values (mg/kg) and GHS toxicity class were recorded. Cytotoxicity against normal cell lines was assessed using the CLC-Pred server (30), with results expressed as probability of activity (Pa) and probability of inactivity (Pi). Values above 0.7 were considered meaningful.

#### 2.2.7 Blood-brain barrier permeability

BBB permeability was specifically evaluated using the B3DB platform (31), which provides a quantitative log BB estimate. Given that GB is a CNS tumor, this was a non-negotiable parameter, a compound that cannot cross the BBB has no practical relevance as a GB therapeutic, regardless of its *in vitro* potency (32).

### 2.3 Cell culture and drug supplementation

Human GB cells (U87MG and LN229) were procured from National Centre for Cell Science (NCCS), Pune, India. The cells were maintained in Dulbecco’s Modified Eagle Medium (DMEM) (11965092, Thermo Fisher Scientific, USA), supplemented with 10% fetal bovine serum (FBS) (10082147, Gibco, Grand Island, NY, USA) and penicillin–streptomycin (A5873601, Thermo Fisher Scientific, USA). They were incubated at 37 °C in a humidified cell culture incubator (Binder, Germany) with 5% CO_2_.

Salidroside (43866, Sigma, St Louis, MO, USA) was dissolved in dimethyl sulfoxide (DMSO) (34869, Sigma, St Louis, MO, USA) to obtain serial dilutions which were used further for dose optimization. While, N-acetyl cysteine (NAC) at a dosage of 100 μM was used as an exogenous antioxidant,100 μM of 4-methylpyrazole (4-MP) was used as a cytochrome P450 2E1(Cyp2E1) inhibitor (33).

### 2.4 Cell viability assay

The cells were supplemented with salidroside at doses of 5, 10, 20, 40, 80 and 160 μM and incubated at durations of 12, 24, 48 or 72 h respectively at each dosage for dose optimization. A separate set of cells cultured with DMSO alone (v/v) at durations of 12, 24, 48 and 72 h served as vehicle control group for each time duration respectively. Cell viability was quantitatively assessed by MTT 3-(4,5-dimethylthiazol-2-yl)-2,5-diphenyltetrazolium bromide (475989, Sigma, St Louis, MO, USA) assay as described previously (34). In brief, U87MG and LN229 cells (~10^5^-10^6^ cells) were plated in 96-well plates and different concentrations of salidroside were added. The cells were incubated at 37 °C for stipulated period of drug treatment. After incubation, 10 µl of MTT (5mg/ml) was added in each well and incubated for 4 h at 37 °C. MTT solution was then aspirated, and the formazan crystals thus formed were solubilized by adding 100 µl of dimethyl sulfoxide (DMSO) in each well. The absorbance was measured at 570 nm in spectrophotometer (BioTek Epoch 2, USA). The cell viability was expressed in percentage considering viability of control group of corresponding duration of exposure to be 100%. The results represent the average of three independent replicates with readings taken in triplicate for each replicate.

### 2.5 Flow cytometry

The quantitative assessment of apoptosis and/or necrosis was assessed using Annexin V/PI kit (APOAF, Sigma, USA) by flow cytometry according to manufacturers’ instructions. Briefly, U87MG and LN229 cells were plated at a density of 1 × 10^6^/ml cells in 6-well plate and then incubated with or without the optimized dosage of salidroside (80 μM) for 24 and 48 h respectively. The control and treated cells were collected and washed with phosphate buffered saline (PBS) (10010023, Thermo Fisher Scientific, USA), resuspended in 500µL of 1x binding buffer and stained with 5µL of Annexin V-FITC conjugate and 10 µL of propidium iodide solution to each cell suspension. The cells were then incubated at room temperature for 10 minutes in dark. The samples were then analyzed to quantify viable, apoptotic (early and late) and necrotic cells using flow cytometer (BD FACS Canto™ II, USA). the quantification was done using CellQuest software. The cells were excited for both Annexin V and PI at 488 nm and emissions for Annexin V-FITC was collected at 530 nm and 610 nm for PI through 610 nm band-pass filter.

### 2.6 Immunofluorescence

U87MG and LN229 cells were plated at 1 × 10^6^ cells per well on lysine-coated coverslips and were allowed to adhere overnight. The treated group were then incubated with 80 μM of salidroside for 24 h and 48 h respectively. The non-treated group without salidroside supplementation served as control. Cells were fixed with 4% paraformaldehyde for 20 min, washed thrice with PBS and then permeabilized with 0.1% triton X 100 in PBS for 15 min at room temperature. The cells were then washed thrice and blocked with 1% BSA for 1 h at room temperature. The cells were incubated with primary antibody Ki67 (9129, Cell Signaling, USA) for 1 h at room temperature, washed thrice with PBS followed by incubation with goat anti–rabbit FITC IgG (H+L) (31635, Invitrogen, USA) for 1 h. After washing thrice with PBS, the cells were counter stained with DAPI and the coverslips were mounted with prolong GOLD anti-fade mounting medium (P36930, Thermo Fisher Scientific, USA). Images were captured using inverted fluorescence microscope (IX85P1ZF, Olympus, Japan). The cell proliferation of all the experimental groups was then analyzed using Image J software (National Institutes of Health, MD, USA). The Ki67 positive cells were counted in five random fields for each sample. To express the fluorescence intensity of Ki67 positive cells, the obtained trichromatic data set was converted into a monochromatic grey-scale and the regions of interest (ROIs) were selected by masking the background. These average grey-scale values from the ROIs of all images were expressed taking control as 100%.

### 2.7 Transwell migration and invasion assay

Migration and invasion assays were performed using 12-wells Trans well inserts (353182, Corning, USA) with a polyethylene terephthalate membrane (8 μm pore size). For the migration assay, U87MG cells (~5x10^5^/ml) with or without salidroside (80 μM) were grown in 6-well culture for 24 and 48 h respectively. The cells were then re-suspended in serum-free media and 200 µl of cells were seeded to the upper chamber. 750 μL of complete medium containing 10% FBS serving as a chemoattractant was added to the lower chamber. The cells were then incubated for 24 h at 37°C. The cells in the upper chamber were gently wiped off with a cotton swab and were washed with PBS. The cells that migrated to the lower surface of the chamber were washed twice in PBS, fixed with 4% formaldehyde for 15 min, permeabilized with 100% methanol for 20 min, and then stained with 0.25% crystal violet for 20 min at room temperature. The number of migratory cells were counted in five randomly selected visual fields for each chamber under light microscope (IX85P1ZF, Olympus, Japan) at 10x objective.

For the invasion assay, the membranes of each upper chamber were coated with 100 μL/well diluted Matrigel (CLS354234, Merck, USA) and incubated at 37 °C overnight for gelling and then reconstituted with 200μl of serum-free medium for 1 h at 37 °C. Unlike the migration assay, cells seeded into the upper chamber were incubated for 48 h instead of 24 h. The subsequent steps remained same as described in the migration assay.

### 2.8 Mitochondrial membrane potential assay

Mitochondria Membrane Potential (MMP) was measured using JC 10 dye supplied with the assay kit (MAK 159, Sigma, St. Louis, USA) according to manufacturers’ instructions. U87MG and LN229 cells were grown to a density of 1x 10^5^ in 96 well black flat bottom plates. Cultures were treated with DMSO (vehicle) or salidroside (80 μM) for 24 and 48 h respectively and incubated separately in 5% CO2 at 37 °C. 5 µl of working solution of JC10 dye was added to the cultures prior to the incubation. 50 µl of JC 10 dye loading solution was then added to each well and incubated in dark for 45 min. 50 µl of assay buffer was added to each plate followed by centrifugation with brakes off at 1000 rpm for 1 min. Fluorescence intensity was then measured at excitation/emission wavelength of 490/525 nm and 540/590 nm.

### 2.9 Cyp2E1 activity

Cyp2E1 activity was estimated based on the concentration of p-nitrocatechol produced according to the protocol suggested by Cederbaum et al. with minor modifications (35). In brief, U87MG and LN229 cells were grown in culture dishes to confluence with and without salidroside for 24 and 48 h respectively. Another subset was treated with 100 µM cyp2E1 inhibitor in the presence of salidroside for 48 h. The cells from all experimental groups were then harvested and protein content in 10 µl of each sample was estimated. 160 µl of reaction mixture containing 100 mM potassium phosphate buffer, pH 7.2, 0.2 mM para-nitrophenol and 1mM NADPH was taken in 1.5 ml centrifuge tubes to which 40 µl of the sample was added and incubated at 37°C in a water bath for 60 min. The reaction was terminated by addition of trichloro-acetic acid (TCA) to a final concentration of 1% followed by mixing and centrifugation at 5000 rpm for 15 min. The supernatant was collected and 20 µl of 10N NaOH was added followed by measurement of optical density at 510 nm. The concentration of p-nitrocatechol was determined from a standard curve and concentration per µg of sample protein was estimated by dividing the values for each sample with protein concentration of the same sample. The experiment was run in triplicates for each group.

### 2.10 NADP+/NADPH assay

NADP/NADPH ratio was assessed using assay kit (MAK479, Sigma, St. Louis, USA) as per manufacturers’ instructions. The kit utilizes a glucose dehydrogenase cycling reaction to measure total NADP+/NADPH content and NADPH concentration separately to allow calculation of the ratio. The assay kit utilizes a method where NADP+ is reduced to NADPH that reduces the formazan dye further resulting in a colorimetric change that is measured spectrophotometrically (BioTek Epoch 2, USA) at 565 nm. In brief, U87MG and LN229 cells (~10^5^) treated or untreated with salidroside and DMSO (vehicle) were washed in ice-cold PBS and homogenized in extraction buffer supplied with the kit. The extract was heated for 60 ^0^C for 5 min and then assay buffers and working reagent were added for further analyses. The ratio of NADP+/NADPH was calculated by extrapolating the values of NADP as standard.

### 2.11 Quantitative real time PCR

Total RNA extraction using Trizol reagent (15596026, Invitrogen, USA) and real time PCR were performed as described previously (36). In brief, U87MG and LN229 cells were grown with and without salidroside (80 μM) for 24 and 48 h respectively, and total RNA was extracted. cDNA was synthesized by reverse transcription using cDNA synthesis kit (205311, QuantiTect Reverse Transcription Kit, QIAGEN, USA) according to manufacturers’ instructions. cDNA was used as a template for PCR amplification using previously published Ngb primer sequence and GAPDH as loading control. The forward primer 5’ TGGAAGACCTGTCCTCACTG 3’ and reverse primer 5’ GAGCAGAGACTCACCCACTG 3’ was used for amplification of Ngb. The forward and reverse primers for GAPDH were 5’ CATCAATGGAAATCCCATCA 3’ and 5’ TTCTCCATGGTGGTGAAGAC 3’ respectively (37). SYBR Green qPCR Kit (208052, QuantiNova SYBR Green PCR Kit, QIAGEN, USA) was used for PCR amplification. The qPCR amplification cycle comprised of: initialization at 95 °C for 5 min 39 cycles of denaturation at 95 °C for 30 s, annealing at 59 °C for 15 s and extension at 72 °C for 30 s. Data were analyzed using the 2−△△Ct method. The experiment was run in triplicates for each group.

### 2.12 Subcellular fractionation and western blot analysis

U87MG and LN229 cells of all experimental groups were washed with PBS and incubated with trypsin for 5 min for subcellular fractionation as described previously (38). The cell suspension was centrifuged at 3000 rpm for 5 min, and the resulting pellet was washed with ice-cold PBS. The cells were then resuspended in homogenization buffer (20 mM HEPES, pH 7.4, 10 mM KCl, 2 mM MgCl_2_, 1 mM EDTA, 1 mM EGTA, 1 mM DTT, and protease inhibitor cocktail) and mechanically disrupted by passing the suspension 15-20 times through a 26-gauge needle. The cell lysate was incubated on ice for 20 min and centrifuged at 3000 rpm for 5 min at 4 °C. The pellet fraction thus obtained contained nuclei and cell debris, while the supernatant (S300) contained the cytoplasm, membranes, and mitochondria. The S300 fraction was centrifuged at 13,000 rpm for 5 min at 4 °C. The pellet (P13,000) represented the mitochondrial fraction, and the supernatant (S13,000) contained the cytoplasmic and membrane components. The S13,000 fraction was subjected to ultracentrifugation at 10,000 rpm for 1 h at 4 °C. The supernatant (S100K) was collected as the cytoplasmic fraction.

For whole cell lysates, U87MG and LN229 cells were lysed in the RIPA buffer (R0278, Sigma, St. Louis, USA) in the presence of protease inhibitor cocktail (P8340, Sigma, St. Louis, USA). After incubation for 30 min at 4 °C, the cells were centrifuged at 15,000 rpm for 30 min at 4 °C and the supernatant was aspirated for further protein expression studies. The whole cell lysates and the cytoplasmic fraction (S100K) were quantified for protein using Bradford assay (B6916, Sigma, St. Louis, USA). Equal amounts of protein were then resolved on 10% SDS PAGE, transferred onto polyvinylidene difluoride (PVDF) (IPVH00010, Merck, Germany) membranes, blocked with 5% BSA in Tris-buffered saline (TBS; 25 mM Tris, 2.6 mM KCl, 0.14 M NaCl, pH 8.0) for 1 h at room temperature. The membranes were then washed thrice with TBS Tween (TBST) (Tween, 0.01 mol/L PBS, pH 7.4) and probed for desired proteins. The membranes were incubated with specific primary antibody overnight at 4 °C. The following antibodies were used- neuroglobin (1:1000, PAA606Hu01, cloud clone corp, China), cleaved caspase 3 (1:1000, ab32042, Abcam, USA), cytochrome c (1:1000, 4272, Cell Signaling Technology, USA) and β-actin (1:1000, A5441, Sigma, St Louis, USA). The membranes were washed thrice with TBST and then incubated with secondary antibodies (31460, HRP-conjugated goat anti-rabbit antibodies, Invitrogen, USA) for 2 h at room temperature. The protein bands were detected using the chemiluminescent peroxidase substrate (CPSOC, Sigma, St Louis, USA) and the images were captured on chemiDoc (BIO-RAD, USA). For densitometric analysis, the blots were scanned and intensities of each band was quantified using ImageJ software and normalized to β-actin that served as loading control.

### 2.13 Lipid peroxidation

The mitochondrial fraction obtained from differential centrifugation was gently resuspended in the Tris–HCl buffer and stored on ice until further analysis. Lipid peroxidation of the mitochondrial fraction was measured as described previously by Hota et al. (39). Briefly, 750 μL of 20% TCA and 750 μL of 0.67% trichloro-butyric acid (TBA) was added to 250 μL of mitochondrial suspension. The cells of all experimental groups were incubated at 85 °C in a water bath, cooled down at room temperature and centrifuged for 5 min at 2,000 rpm. 200 μL of the supernatant was aspirated to detect absorbance at 531 nm in a spectrophotometer (BioTek Epoch 2, USA). The amount of malondialdehyde (MDA) formed as a by-product was then calculated using the MDA–TBA complex’s molar extinction coefficient, which is 1.56 × 10^5^ cm^2^ mmol−1. The experiment was run in triplicates for each group and values were then expressed in µM/mg of protein.

### 2.14 Reactive oxygen species detection within cells

U87MG and LN229 cells were plated at 1 × 10^6^ cells per well in 6 well plate and were allowed to adhere overnight. The cells were supplemented with 80 μM of salidroside for 24 h and 48 h respectively. The non-treated group without salidroside supplementation served as control. Thereafter, the cells were rinsed thrice with ice-cold PBS. Cells were then supplemented with 10 µM of Carboxy-DCFDA (Sigma Aldrich, 21884, Germany) in serum-free medium and incubated for 30 min at 37 °C in the dark (40). Images were captured using inverted fluorescence microscope (IX85P1ZF, Olympus, Japan). The fluorescence intensity of DCFDA of all the experimental groups was then analyzed using Image J software (National Institutes of Health, MD, USA).

### 2.15 Iron concentration assay

Fe^2+^ and Fe^3+^ iron was assayed colorimetrically from cell lysates using iron assay kit (ab83366, Abcam, USA). Cells of all experimental groups at a concentration of 1x10^5^ were lysed in 100 µL of assay buffer using a sonicator. The samples were centrifuged at 10,000 rpm for 10 min at 4°C and the supernatant was aspirated for further analyses. Samples were run in duplicates with 40 µL of sample being added to each well along with 60 µL of iron assay buffer. 5 µL of iron reducer was added to one of the sample wells to estimate total iron while 5 µL of assay buffer was added to the other well to estimate ferrous iron. Samples and standards were mixed and incubated at 37 °C for 30 min as suggested by the manufacturer. 100 µL of iron probe was added to each well, mixed and incubated in dark for 60 minutes at 37 °C followed by measurement of optical density at 593nm.

### 2.16 Immunohistochemistry

Formalin-fixed, paraffin-embedded tumor tissues were sectioned into 4-µm-thick slices, deparaffinized in xylene, and rehydrated through a graded ethanol series. Antigen retrieval was performed by heating the sections in Tris–EDTA buffer for 3 min using a pressure cooker. Endogenous peroxidase activity was quenched by incubation with 0.3% hydrogen peroxide for 30 min, followed by washing with phosphate-buffered saline (PBS). Non-specific binding was blocked by incubating the sections with 10% skim milk in PBS for 30 min. The sections were then incubated for 1 h at room temperature with an anti-neuroglobin (Ngb) primary antibody (Abcam, Cambridge, MA, USA) diluted 1:300 in PBS. After washing with PBS, the sections were incubated with a peroxidase-conjugated secondary antibody (Pathnsitu Biotechnologies, USA) for 90 min. Immunoreactivity was visualized using a diaminobenzidine (DAB) substrate for 5 min. The sections were counterstained with hematoxylin, dehydrated, and mounted (12) Staining intensity was independently evaluated and scored as follows: Intensity 0- no staining; 1- moderate staining; 2- mild staining; and 3-intense staining and area 0 -no expression, 1- 1-25% of the area, 2- 26-50% of the area, 3-51-75% of the area, 4- 76-100% of the area (41). Immunoreactivity was quantified using an H-score, calculated as the sum of the staining area score and the staining intensity score.

### 2.17 Statistical analysis

Statistical analyses were performed using Graph Pad Prism 8.0. The data were obtained and expressed in terms of mean ± SD. Comparisons across experimental groups were performed using ordinary one-way ANOVA followed by Tukey’s multiple comparisons test as the *post hoc* test, for all analyses except the dose-response curves. For dose-response analyses, two-way ANOVA followed by Tukey’s multiple comparisons test was used to account for the two independent variables (dose and treatment/time). All experiments were performed with five independent biological replicates (n = 5) with each biological replicate performed in triplicate. Adjusted P values of P < 0.05, <0.01, 0.001 and <0.0001 were considered to be statistically significant.

## 3 Results

### 3.1 Neuroglobin is downregulated in GB

To assess the role of Ngb in glioma, Ngb expression was assessed in glioma patients at different tumor stages and in normal brain tissue by immunohistochemical staining. Quantitative assessment using the H-score, calculated as the sum of staining area and intensity, demonstrated significantly lower scores of Ngb in GB samples, indicating decreased immunoreactivity with increasing tumor grade (**Figure 1C**). These findings suggest a grade-dependent downregulation of Ngb, with minimal levels observed in GB. These findings were consistent with the immunoblotting results. Ngb protein levels were examined in glioma tissue samples across different tumor grades (n = 5 per grade) and compared against control tissues (n = 5). A progressive decline in Ngb protein expression was observed with advancing tumor grade, suggesting an inverse relationship between Ngb levels and glioma severity. Collectively, these findings indicate that downregulation of Ngb may serve a functionally significant tumor-suppressive role in the progression of glioma.

**Figure 1:**
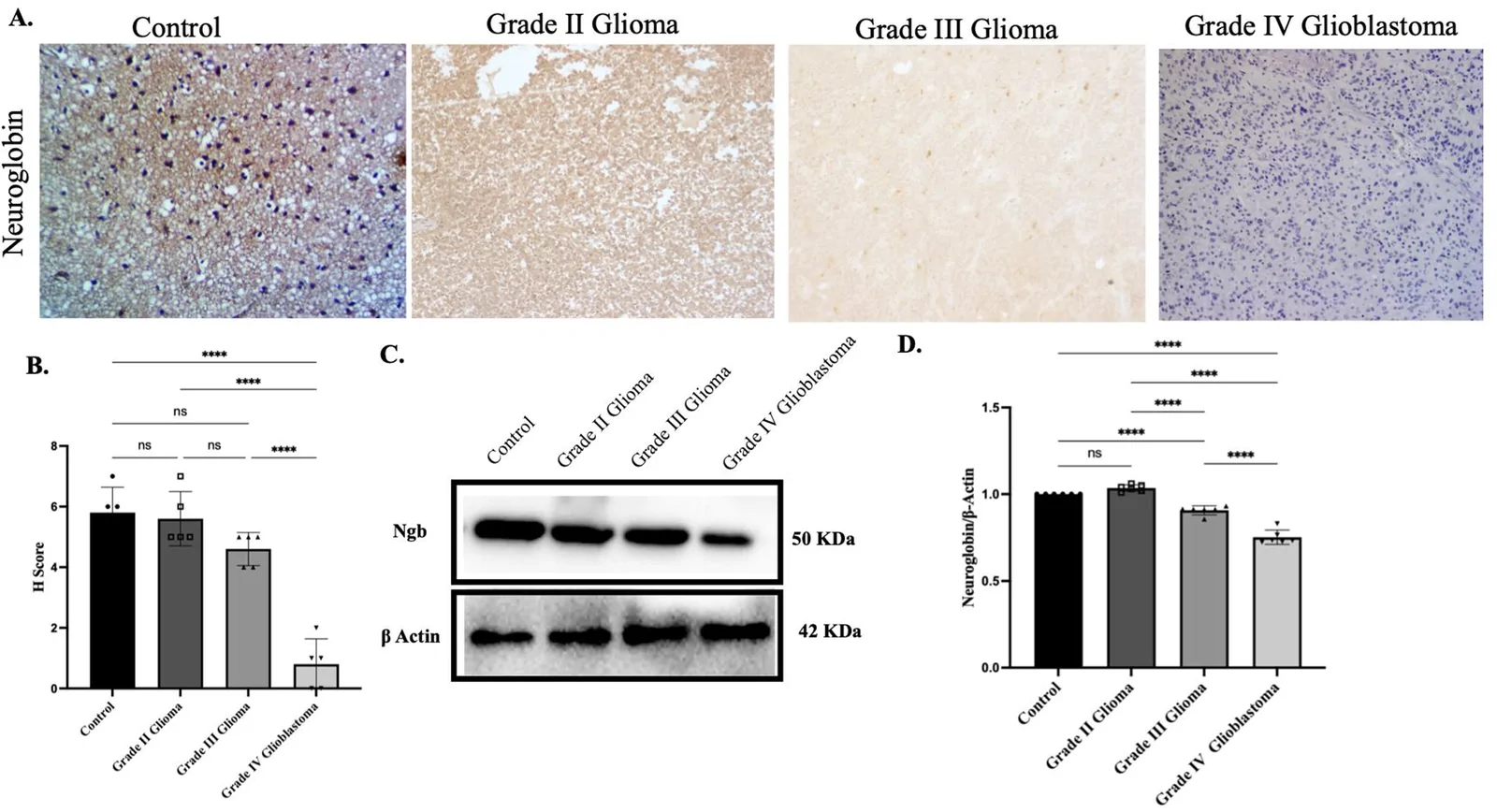
Neuroglobin is downregulated in glioblastoma. **(A)** Representative IHC images of Ngb (20x) in different grades of glioma **(B)** H score of Ngb Immunohistochemistry. **(C)** Relative protein expression of Ngb in patient samples as detected by immunoblotting. β- actin was considered as loading control **(D)** Quantitative protein fold change of Ngb in patient samples normalized with β-actin. Graph represents mean ± SD. ****P <0.0001 was considered significant when compared with control and study groups. versus control and study groups; ns, non-significant.

### 3.2 Salidroside–Ngb interaction is influenced by redox state

#### 3.2.1 Binding energies and pose distribution

Molecular docking was performed to evaluate the interaction of salidroside with Ngb in its ferrous (Fe²^+^) and ferric (Fe³^+^) states (**Figures 2A, B**). Salidroside demonstrated slightly more favourable binding energies toward the Fe²^+^ form compared to Fe³^+^, with best docking scores of −7.04 kcal/mol and −6.82 kcal/mol, respectively. The Fe²^+^ state also generated a greater number of docked conformations, suggesting increased conformational accessibility within the reduced binding pocket. Energy distribution analysis indicated a broader cluster of low-energy poses for the Fe²^+^ complex, whereas the Fe³^+^ complex showed a narrower distribution, consistent with comparatively reduced binding flexibility (**Table 2**).

**Figure 2:**
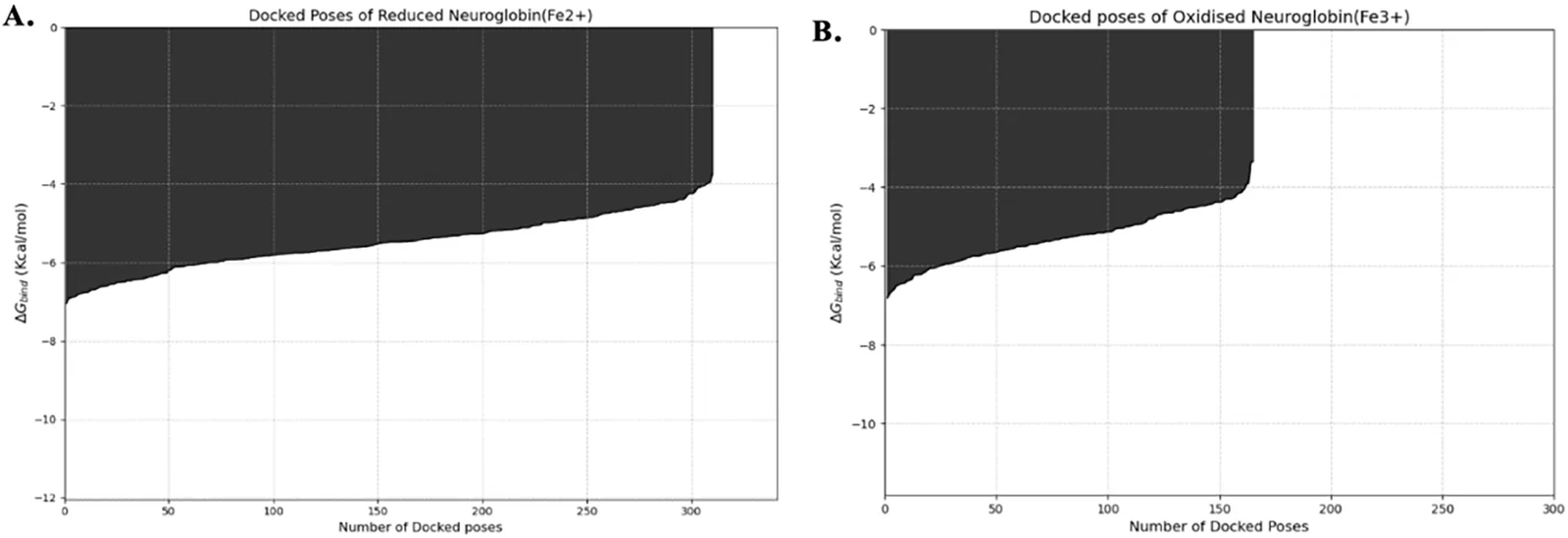
Docked pose energy distributions for salidroside with oxidized and reduced Ngb **(A)** Binding energy distribution plots for the Ngb (Fe2^+^)–Salidroside complex (left) (B) Ngb (Fe3^+^)–Salidroside complex (right), from 2000 docked conformations. Note the broader, more densely populated low-energy cluster for the Fe²^+^ complex, indicating a more conformationally stable binding mode.

**Table 2:** Predicted binding parameters of salidroside with ferrous (Fe²^+^) and ferric (Fe³^+^) state of Ngb through molecular docking analysis.

Neuroglobin form	Number of poses	Lowest binding energy	Final intermolecular energy	vdW + Hbond + desolv energy	Electrostatic energy
Fe²^+^ (Reduced)	310	-7.04	-10.02 kcal/mol	-9.36 kcal/mol	-0.66 kcal/mol
Fe³^+^ (Oxidized)	165	-6.82	-9.81 kcal/mol	-9.20 kcal/mol	-0.61 kcal/mol

#### 3.2.2 Binding pose characteristics

Top-ranked docking poses for the Fe²^+^ complex (**Figure 3A**) showed consistent ligand positioning near the heme-binding region of Ngb with minimal variation in binding energy. In contrast, the Fe³^+^ complex (**Figure 3B**) exhibited greater variability in ligand orientation and slightly weaker binding energies (**Supplementary Table 1**).

**Figure 3:**
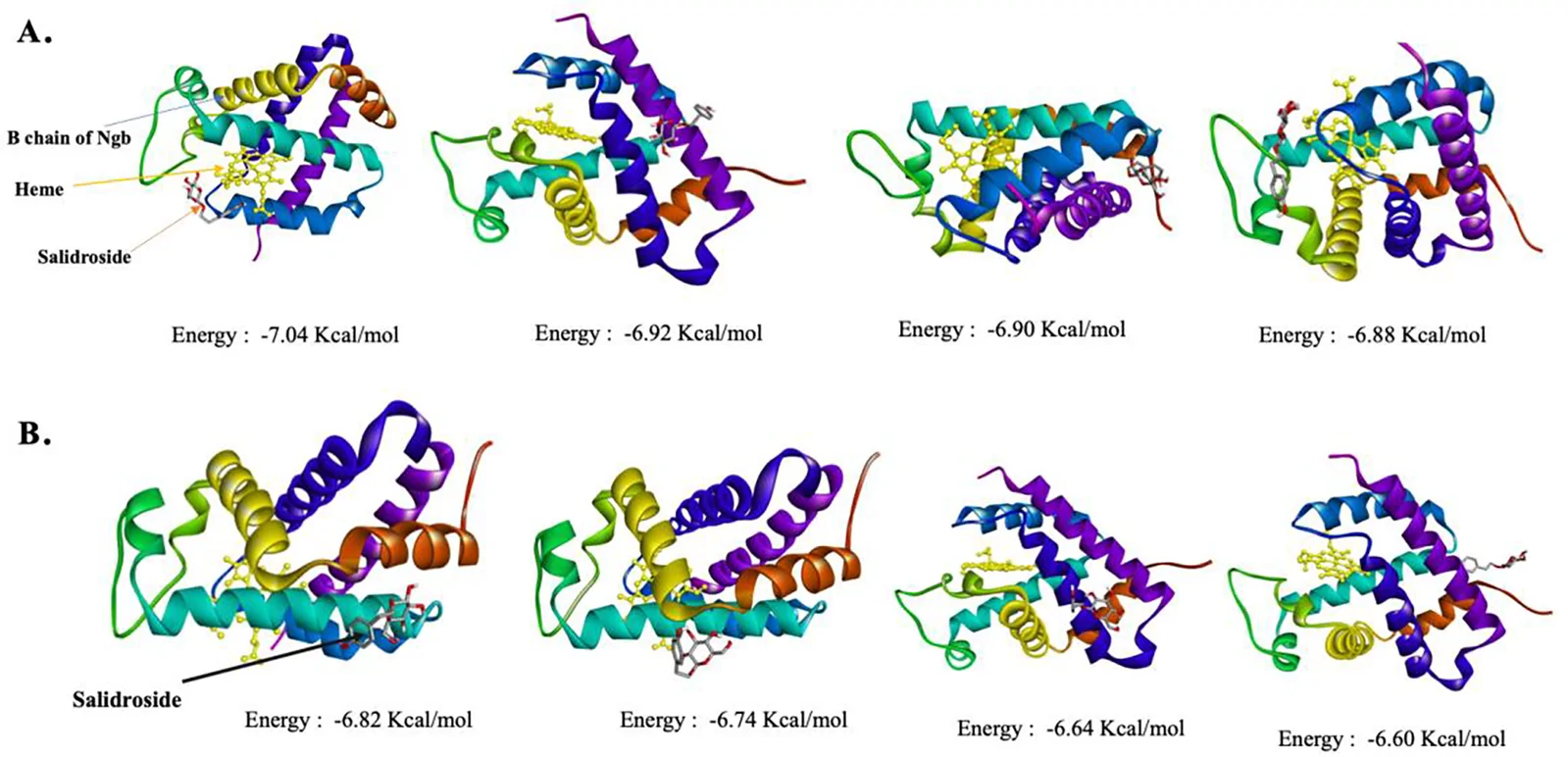
Three-dimensional docking poses of salidroside within the Ngb active site. **(A)** Top four docked conformations of salidroside in the Fe²^+^ (reduced) Ngb active site, with binding free energies of −7.04, −6.92, −6.90, and −6.88 kcal/mol. Ribbon representation; Salidroside shown in yellow stick. **(B)** Top four poses in the Fe³^+^ (oxidized) Ngb active site, with binding free energies of −6.82, −6.74, −6.64, and −6.60 kcal/mol.

### 3.3 Molecular interaction

Interaction profiling revealed that salidroside forms multiple hydrogen bonds within the Ngb binding pocket, with a higher number observed in the Fe²^+^ complex compared to Fe³^+^ (**Supplementary Table 2**). Key interacting residues in the Fe²^+^ state (**Figure 4**) was located within the heme-binding region, whereas interactions in the Fe³^+^ state were more peripheral. Overall, these findings indicate that the interaction of salidroside with Ngb may be influenced by its redox state, with a tendency toward more favorable binding in the reduced (Fe²^+^) form. 

**Figure 4:**
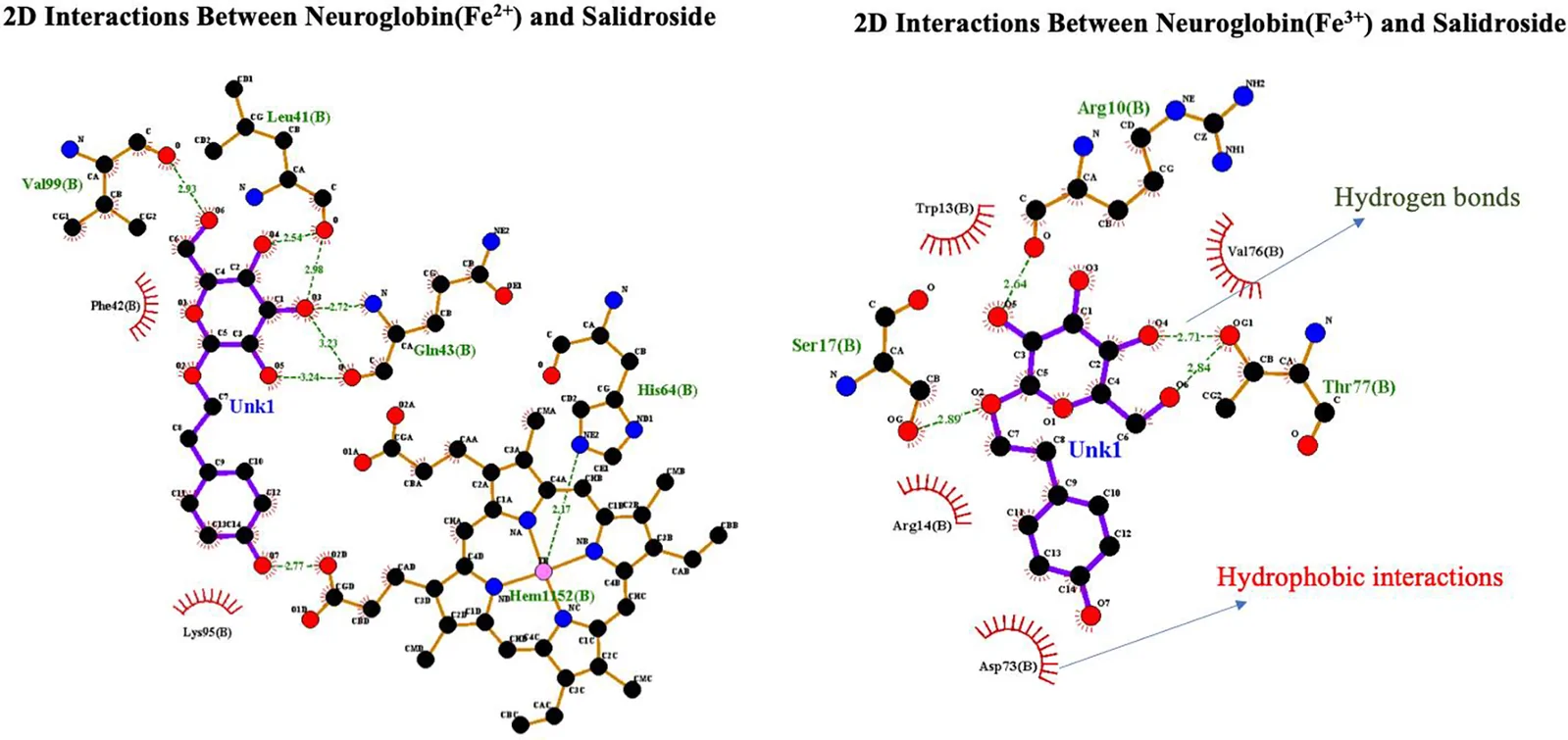
Two-dimensional protein–ligand interaction maps comparing salidroside binding across Ngb redox states (LigPlot^+^) (https://www.ebi.ac.uk/thornton-srv/software/LigPlus/). Hydrogen bonds are shown as dashed green lines with distances in Å; hydrophobic contacts are indicated by arc representations. Note the greater number, shorter distances, and deeper heme-pocket localization of hydrogen bonds in the Fe²^+^ complex relative to the Fe³^+^ complex, and the unique direct Fe coordination (2.17 Å) present only in the reduced state.

### 3.4 Predicted cytotoxicity toward normal cells

In-silico cytotoxicity prediction indicated a low probability of salidroside toxicity toward most normal cell types. While moderate activity was predicted in select fibroblast-derived cell lines, overall results suggest a favorable safety profile; however, these predictions require experimental validation (**Supplementary Table 4**).

### 3.5 ADMET and drug-likeness properties

Salidroside exhibited physicochemical properties consistent with drug-like molecules, including moderate molecular weight, high solubility, and predicted oral absorption. The compound satisfied major drug-likeness criteria with no significant structural alerts. Predicted metabolism suggests minimal interaction with major CYP450 isoforms, indicating a low likelihood of metabolic drug–drug interactions (**Supplementary Table 3**; **Supplementary Figure 1**). However, these findings are based on computational models and should be interpreted cautiously.

### 3.6 Pharmacokinetic and safety predictions

Pharmacokinetic modelling indicated moderate tissue distribution and acceptable systemic exposure. Toxicity prediction platforms suggested low acute toxicity and the absence of major safety liabilities such as hepatotoxicity or cardiotoxicity (**Supplementary Figures 2A, B**).

### 3.7 Blood–brain barrier permeability

Salidroside was predicted to be capable of crossing the blood–brain barrier, with a logBB value (**Supplementary Figure 2C**) within the range considered permissive for CNS exposure. This suggests potential relevance for central nervous system applications, although *in vivo* validation is required.

### 3.8 Salidroside reduces cell viability in U87MG and LN229 cells

MTT assay was performed to assess the cytotoxic effect of salidroside on U87MG and LN229 cells by measuring the metabolic activity of the cells. The cells were treated with different concentrations of salidroside (0, 5,10, 20, 40, 80, 160 µM) for 12, 24, 48 and 72 h. the MTT assay revealed a dose-dependent cytotoxic effect of salidroside on U87MG and LN229 cells as illustrated in **Figure 5A, B** respectively. When compared with control group, salidroside treatment groups exhibited time-dependent (12, 24, 48 and 72 h) and dose-dependent (0-160 µM) reduction in U87MG and LN229 cells. While the cells treated with 80 µM of salidroside for 48 and 72 h exhibited ~60% inhibitory effect (P < 0.001), cells treated with 80 µM of salidroside for 24 h displayed nearly 75% viability when compared with control.

**Figure 5:**
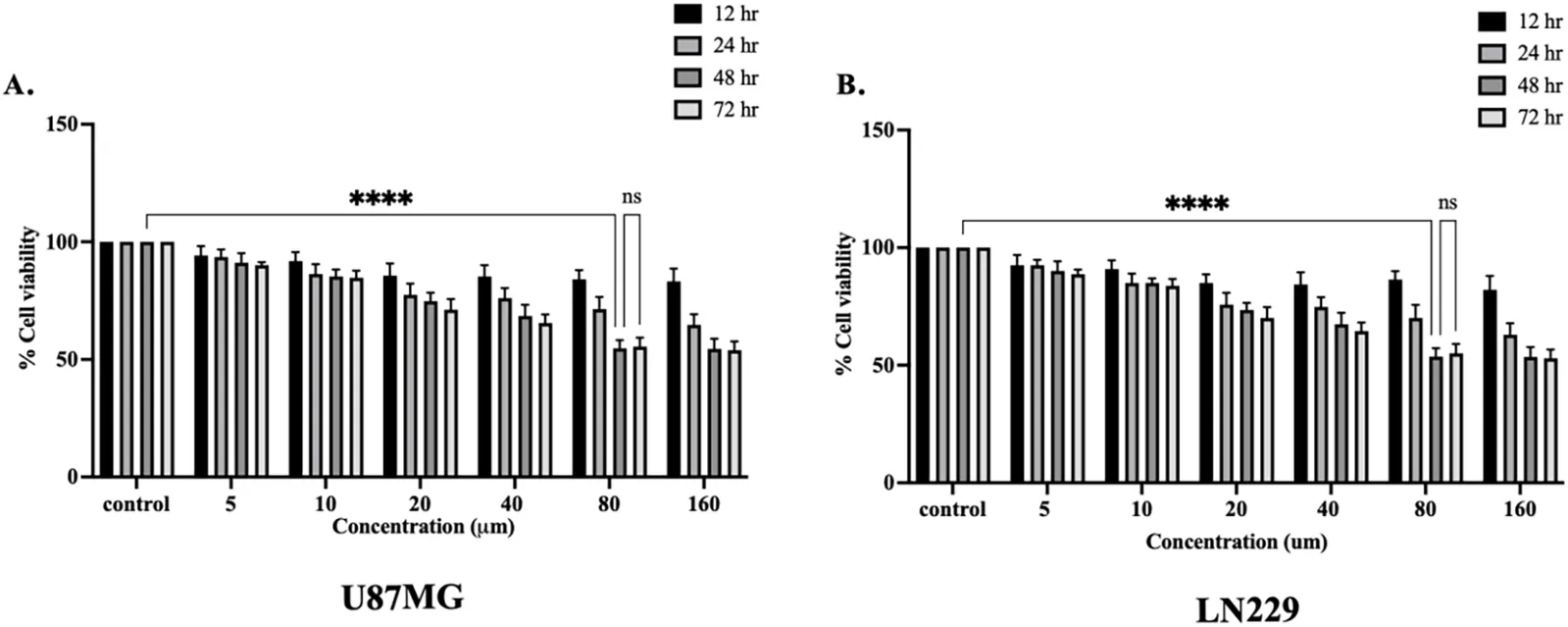
Salidroside inhibits growth of U87MG and LN229 cells. **(A, B)** Effect of salidroside on cell viability as measured by MTT assay in U87MG and LN229 cells. ****P is <0.0001 was considered significant when compared with control and study groups. ns, non-significant.

Based on our dose-response viability data, which showed that 80 μM significantly reduced cell viability in both U87MG and LN229 cells, the 80 μM concentration was chosen. Higher concentrations (up to 160 μM) did not result in a further significant reduction in viability, suggesting that the cytotoxic and anti-proliferative effect had plateaued by this dose. Therefore, subsequent experiments were performed using 80 µM of salidroside for 24 (shorter duration) and 48 h (longer duration).

### 3.9 Salidroside inhibits proliferation and triggers apoptosis in U87MG and LN229 cells

Ki67, a nuclear non-histone protein expressed ubiquitously in proliferating cells (G1, S, G2 and M) is often used as a biomarker to assess cell proliferation in various cancers (41). The anti-proliferative effects of salidroside were thus evaluated by immunofluorescence of Ki67 in U78MG and LN229 cells. DAPI was used as a counter stain. The control cells (non-treated cells) exhibited higher Ki67 expression when compared with salidroside supplemented groups (**Figures 6A–D**). 80µM of salidroside treatment for 24 and 48 h inhibited the proliferation of U87MG and LN229 cells significantly (p<0.0001). No significant difference was observed in vehicle groups of 24 and 48 h duration. The results obtained were consistent to MTT assay and indicate the potential of salidroside for GB tumor suppression.

**Figure 6:**
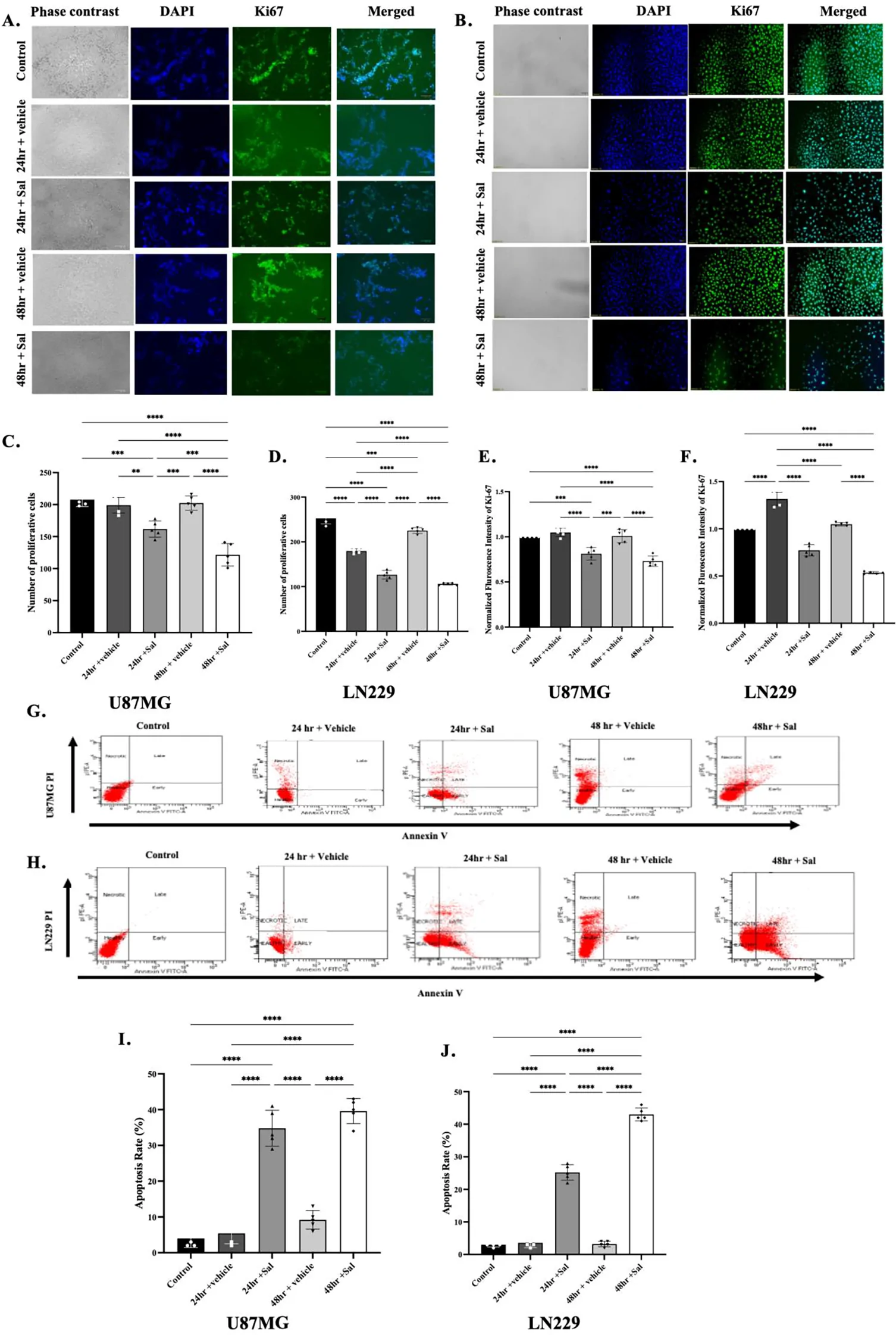
Salidroside inhibits cell proliferation and triggers early apoptosis in U87MG cells. **(A, B)** Representative phase-contrast and fluorescence microscopic images of U87MG cells and LN229 cells incubated with salidroside (80µM) at 24 and 48 h of incubation (10x). **(C)** The graph depicts number of proliferative cells detected by immunostaining with DAPI and Ki67 **(D)** The graph depicts number of proliferative cells detected by immunostaining with DAPI and Ki67 in LN229 cells. **(E, F)** The graph depicts fluorescence intensity as detected by dual staining with DAPI and Ki67 in U87MG and LN229 cells**. (G, H)** Representative flow cytometry images of U87MG cells and LN229 cells supplemented with and without salidroside (80µM). **(I-J)** Percentage of apoptotic cells in U87MG and LN229 cells. The percentage of apoptotic (right lower quadrant, early apoptotic cells; right upper quadrant and late apoptotic cells) was analyzed using BD FACS diva 9.0 software. Data are expressed as mean ± standard deviation (SD). P values indicate significant differences compared with control or vehicle group respectively. ****P is <0.0001 , ***P is <0.001, **P is <0.01 was considered significant when compared with control and study groups. Sal, Salidroside.

Further, in order to determine whether induction of glioma cell apoptosis is related to inhibition of proliferation, Annexin V/PI staining was performed using flow cytometry. Annexin V/PI staining is a valuable tool to assess the mechanism of cell death by analyzing the proportion of cells in each quadrant (viable, early apoptotic, late apoptosis and necrosis). As depicted in **Figures 6G–J** treatment with 80µM of salidroside for 24 h resulted in 25.2% of U87MG to be apoptotic and 36.4% of LN229 to be apoptotic. The cells supplemented with salidroside (80µM) for 48 h depicted 39.48% apoptotic U87MG cells and 45.4% apoptotic LN229 cells as shown in **Figures 6G–J**. The results were found to be statistically significant when compared with the vehicle and control groups respectively (p<0.0001).

### 3.10 Salidroside inhibits migration and invasion in U87MG cells and LN229 cells

To further investigate the anti-glioma potential of salidroside, trans-well migration and invasion assays were performed in U87MG cells and LN229 cells. As depicted in **Figures 7A, B**, 80 µM of salidroside supplementation for 24 h inhibited the migration of U87MG cells and LN229 cells. Notably, the numbers of migrated cells were significantly reduced in those cells which were treated with 80 µM of salidroside for 48 h indicating a time-dependent anti-glioma effect (p<0.0001). Further, the groups treated with vehicle alone for 24 and 48 h respectively did not show any significant difference in the pattern of migratory cells when compared with salidroside supplementation. The quantitative data depicted in **Figure 7C** indicated 62.4% reduction in migration at 24 h and 60.2% reduction at 48 h in U87MG cells (42). The quantitative data depicted in **Figure 7D** indicated 64.4% reduction in migration at 24 h and 56.8. % reduction at 48 h in LN229 cells. We also evaluated the anti-invasive effect of salidroside in U87MG and LN229 cells using matrigel-coated filter after 24 and 48 h supplementation. The panels depicted in **Figure 7G** indicate 60.2% reduction in invasion at 24 h and 58.6% reduction at 48 h of salidroside supplementation considering controls to be 100% in U87MG cells. The panels depicted in **Figure 7H** indicate 57.4% reduction in invasion at 24 h and 47% reduction at 48 h of salidroside supplementation considering controls to be 100% in LN229 cells. The results thus indicate that salidroside considerably inhibited cell invasion as evidenced by the ability of salidroside-treated U87MG and LN229 cells to traverse the matrigel-coated layer when compared with control group.

**Figure 7:**
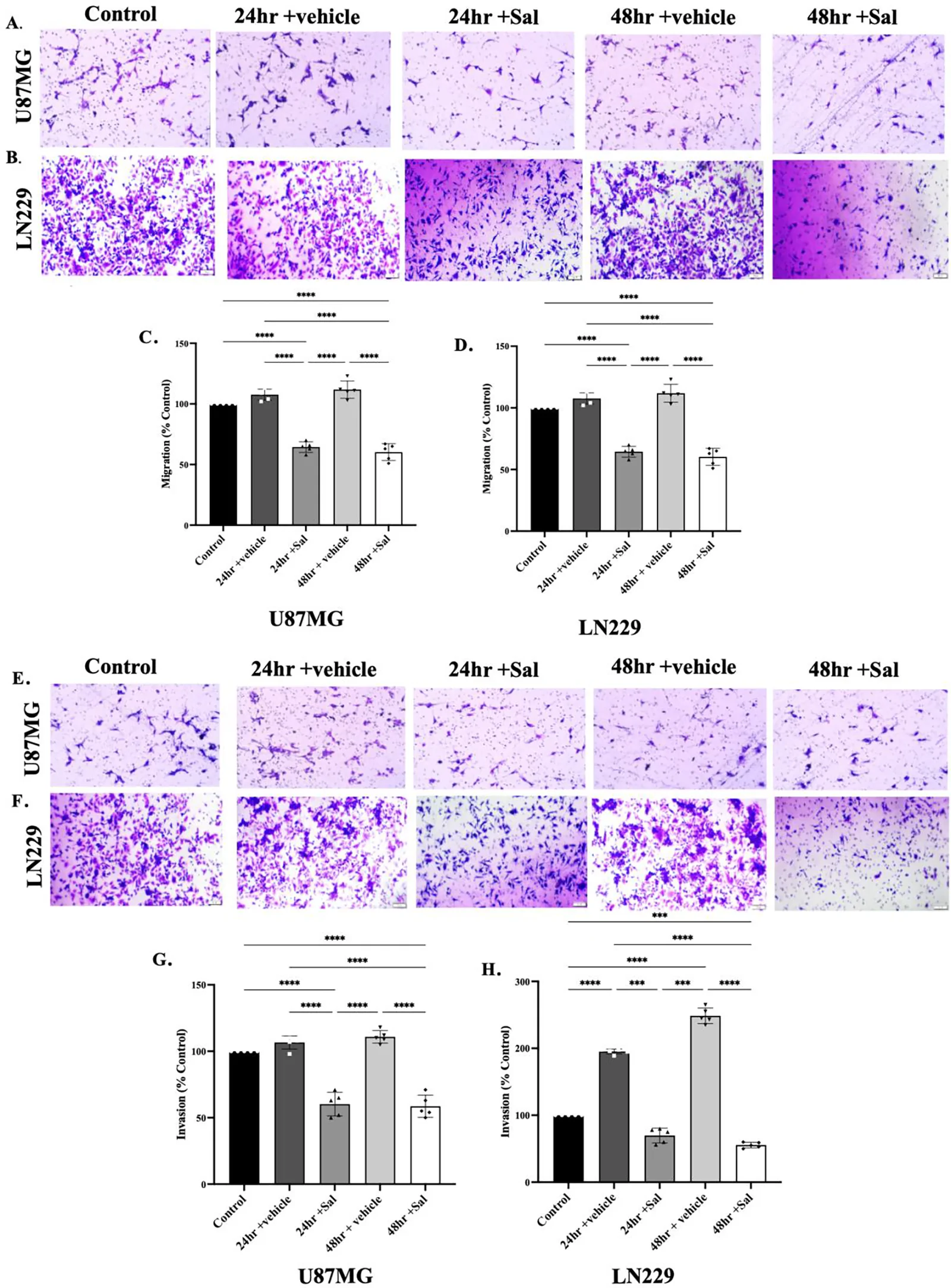
Salidroside inhibits migration and invasion in U87MG and LN229 cells. **(A)** Representative images of U87MG cells and LN229 cells **(B)** Trans-well migration assay with crystal violet staining (10x) representing reduced migration of cells at 48 h of incubation with 80µM of salidroside. **(C, D)** Migration in U87MG and LN229 cells. **(E, F)** Representative images of U87MG and LN229 cells. **(G, H)** Trans-well invasion assay with crystal violet staining (10x) in U87MG and LN229 cells. ****P is <0.0001 , ***P is <0.001 was considered significant when compared with control and study groups. Sal, Salidroside.

### 3.11 Salidroside increases ROS production and reduces mitochondrial membrane potential promoting lipid peroxidation in U87MG and LN229 cells

To determine the role of ROS in salidroside induced apoptosis, the levels of ROS generation were assessed using fluorescence sensitive probe C-DCFDA. The results as depicted in **Figures 8A–C** reveal increased ROS generation on supplementation with salidroside for 24 and 48 h when compared with control. Treatment with ROS scavenger, NAC not only almost abolished salidroside induced apoptosis, but also partly reversed the decrease of cell viability in LN229 and U87MG cells (data not shown). Since, mitochondria play a vital role in inducing caspase-dependent/independent apoptosis by releasing various pro-apoptotic proteins, primarily cytochrome c, Smac/Diablo, Apoptosis-inducing factor (AIF) etc mitochondrial membrane potential (MMP) (ΔψM) was also assessed. MMP is also essential for sustaining oxidative phosphorylation and production of adenosine triphosphate (ATP) by actively pumping hydrogen ions from the matrix into the intermembrane space, (43). (43) Depolarization of MMP and lipid peroxidation has been shown to precede cell death in several models (44, 45) displaying characteristic feature of apoptosis. To further explore the mechanism of action of salidroside in glioma cells, we examined MMP using JC-10 dye and lipid peroxidation using MDA assay.

**Figure 8:**
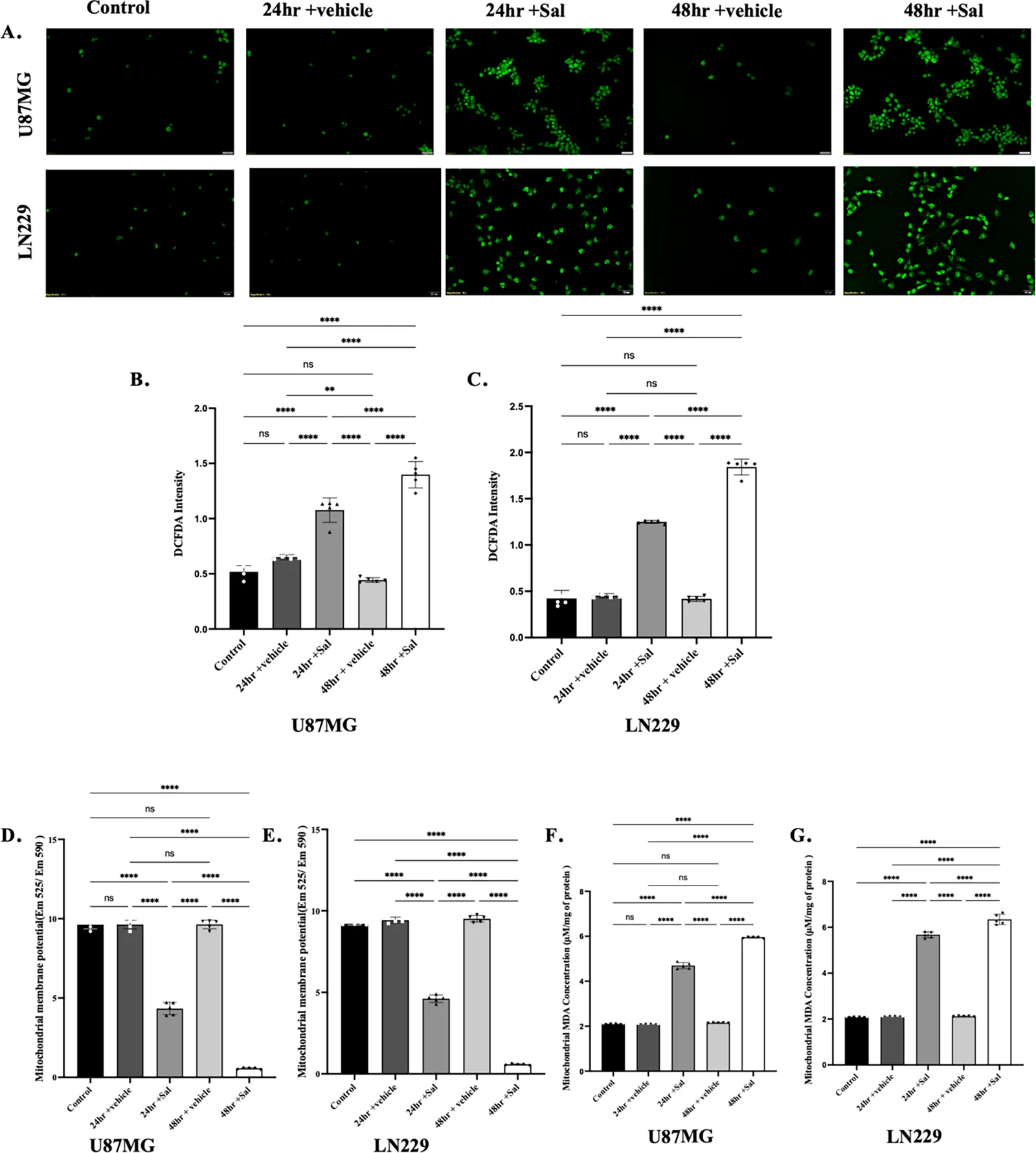
Salidroside reduces MMP and promotes lipid peroxidation and intracellular ROS generation in U87MG and LN229 cells. **(A–C)**. Representative images of U87MG and LN229 of DCFDA staining (20x) representing increased ROS production post salidroside supplementation for 48 h when compared with control. **(D, E)** MMP assessed by JC-10 dye in U87MG and LN229 cells. (**F, G)** Lipid peroxidation by MDA assay in U87MG and LN229 cells with and without salidroside treatment. ****P is <0.0001, **P is <0.01 was considered significant when compared with control and study groups. ns, non-significant. Sal, Salidroside.

JC-10 is a cationic, liophilic dye, exhibiting greater solubility than JC-1 dye, forming reversible red-fluorescent aggregates (λex = 540/λem = 590 nm) in the cells with polarized mitochondrial membrane, while in apoptotic cells, it returns to its monomeric green fluorescent form (λex = 490/λem = 525 nm) indicating MMP collapse. We determined MMP in salidroside treated and non-treated groups using JC-10 probe in U87MG cells. Our results displayed significant reduction in MMP (>95.5%) in cells treated with 80µM of salidroside for 48 h when compared with control as indicated in **Figures 8D, E**. The cells treated with DMSO serving as vehicle groups did not show any significant change in MMP when compared with controls. These results further validate the pro-apoptotic action of salidroside by promoting MMP collapse, increasing mitochondrial membrane permeability and releasing pro-apoptotic proteins.

Lipid peroxidation has been widely recognized as a convergent mediator of multiple regulated cell death pathways, encompassing necroptosis, pyroptosis, apoptosis, and ferroptosis. While the present study demonstrated evidence of lipid peroxidation associated oxidative stress culminating in apoptosis, a formal mechanistic interrogation of ferroptosis was beyond the scope of the current investigation and warrants dedicated evaluation in future studies. Further, lipid peroxidation significantly influences the initiation and regulation of apoptotic mechanisms through oxidative modification of poly-unsaturated fatty acid (PUFA) rich cell membranes, in particular, mitochondria. Due to the important role of lipid peroxidation in several diseases and cancers, intense efforts are being taken to identify and develop compounds that could increase the production of peroxides thereby providing a favorable therapeutic strategy to suppress tumor growth. We explored the potency of salidroside in ameliorating lipid peroxidation by quantifying the end products of lipid peroxidation. Treatment of U87MG and LN229 cells with 80 µM of salidroside significantly increased lipid peroxidation in a time-dependent manner (p<0.0001) when compared with control group as depicted in **Figures 8F, G**.

### 3.12 Salidroside increases CYP2E1 activity and reduces the NADPH/NADP^+^ ratio

Brain drug biotransformation and bioavailability is greatly impacted by the cytochrome P450 (CYP) enzymes expression. CYP enzymes, chemically oxidize or reduce drugs using iron and NADPH as a cofactor (46). Numerous findings imply the controversial role of CYP2E1, one of the most potent CYP450 isoforms, in producing intracellular reactive oxygen species (ROS), in the control or progression of tumor via inflammatory responses, carcinogenesis in many organs, enhancing proliferation and invasion while inhibiting apoptosis in gastric cancer cells (47, 48). The present study, thus, explored CYP2E1 activity in response to salidroside supplementation in the proliferation or apoptosis in U87MG and LN229 cells. The results as depicted in **Figures 9A, B** revealed increase CYP2E1 levels post salidroside treatment for 24 and 48 h respectively when compared with control cells without salidroside (P<0.0001). Since, iron and NADPH act as a cofactor for CYP2E1 activity, we posit to assess the (Fe^2+^/Fe^3+^) and NADPH/NADP^+^ ratio in glioma cells. The results display significant increase in Fe^2+^/Fe^3+^ ratio on supplementation with salidroside for 24 and 48 h (**Figures 10A, B**, p<0.0001). The control and vehicle groups showed no statistical difference. A concomitant increase in NADPH oxidation was also observed as depicted in **Figures 9C, D** (p<0.0001).

**Figure 9:**
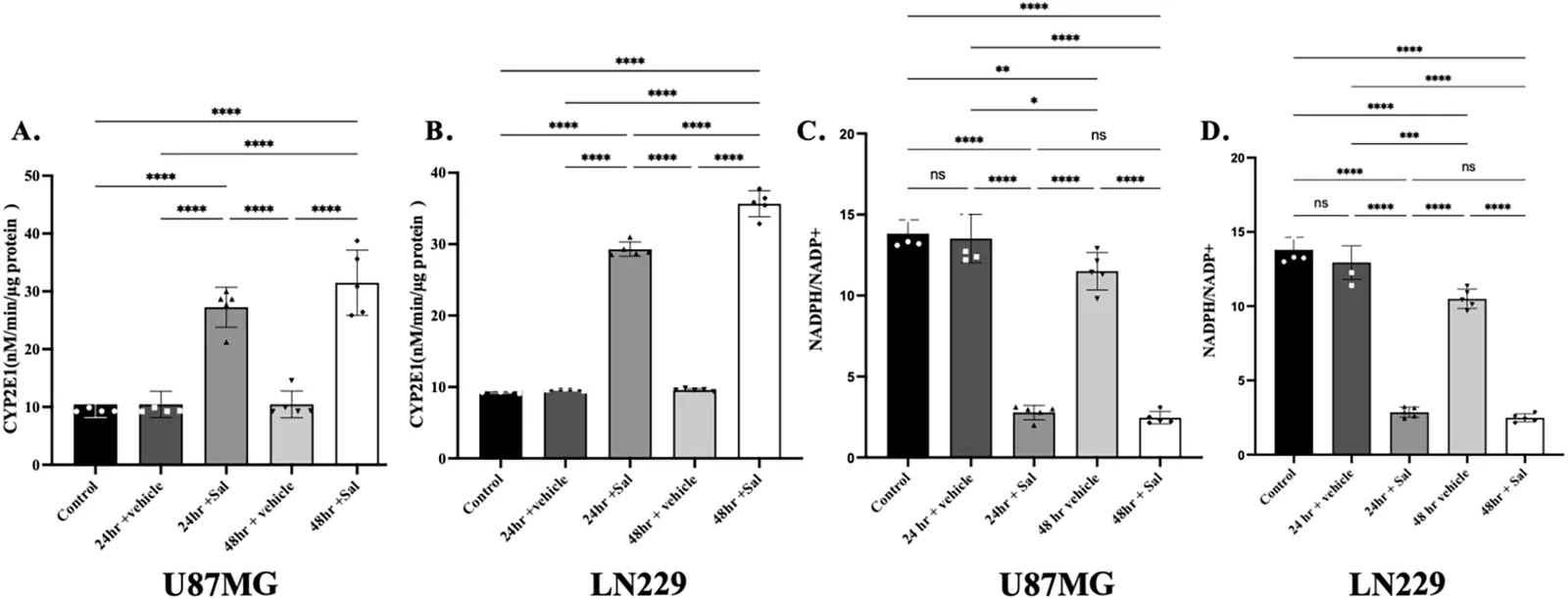
Salidroside mediated increase in CYP2E1 activity and reduction in NADPH/NADP+ ratio contributes to redox imbalance, oxidative stress and lipid peroxidation in U87MG and LN229 cells. **(A, B)** CYP2E1 activity expressed in nM/µg of protein with and without salidroside in U87MG and LN229 cells for 24 and 48 h respectively. **(C, D)** NADPH/NADP+ ratio in U87MG and LN229 cells. Graph depicts mean ± SD ****P <0.0001 ,***P<0.001, **P <0.01, *P <0.05 was considered significant when compared with control and study groups. ns, non-significant. Sal, Salidroside.

**Figure 10:**
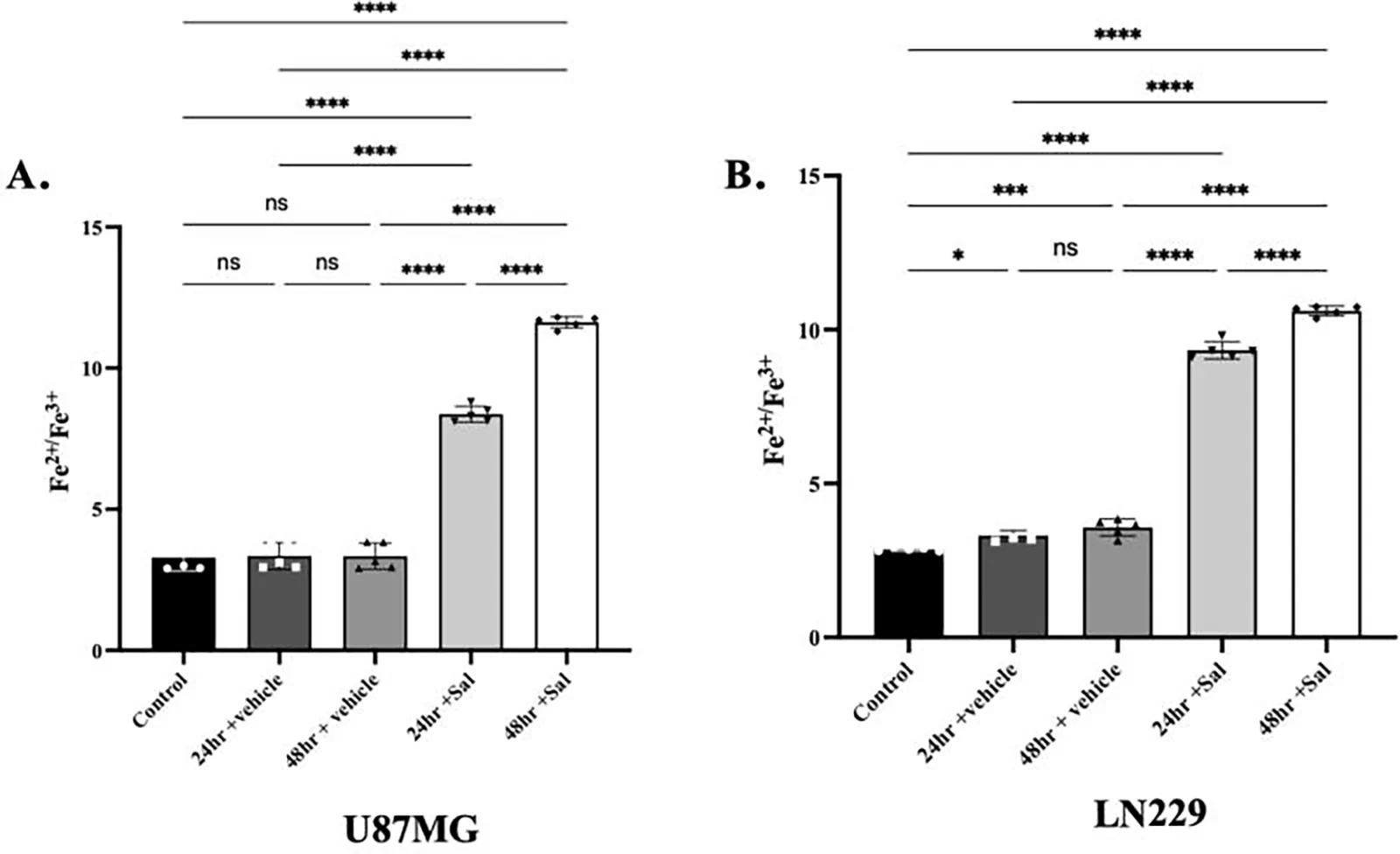
Salidroside increases ferrous (Fe^2+^) to ferric (Fe^3+^) ratio in U87MG and LN229 cells **(A, B)** Ferrous (Fe^2+^) to Ferric (Fe^3+^) ratio in U87MG and LN229 cells. **(B)** Ferrous (Fe^2+^) to Ferric (Fe^3+^) ratio LN229 cells. ****P <0.0001, ***P <0.001, *P <0.05 was considered statistically significant when compared with control and study groups. Sal, Salidroside.

### 3.13 Cyp2E1 activity and mitochondrial ROS mediated oxidation of Ngb could result in altered ferrous/ferric ratio and trigger early apoptosis in salidroside treated U87MG and LN229 cells

To explore the regulatory role of Ngb in glioma progression as well as the efficacy of salidroside in modulating the expression of Ngb, we investigated the mRNA as well as protein expression of Ngb. Interestingly, no change was observed in mRNA levels of Ngb in cells supplemented with salidroside (80µM) for 24 and 48 h as depicted in **Figure 11E**. However, we observed significant increase in the protein levels of Ngb on salidroside supplementation for 24 and 48 h (**Figures 11A, B**). These data suggest that salidroside mediated post translational upregulation of Ngb could be a contributing factor for the observed anti-cancerous effect.

**Figure 11:**
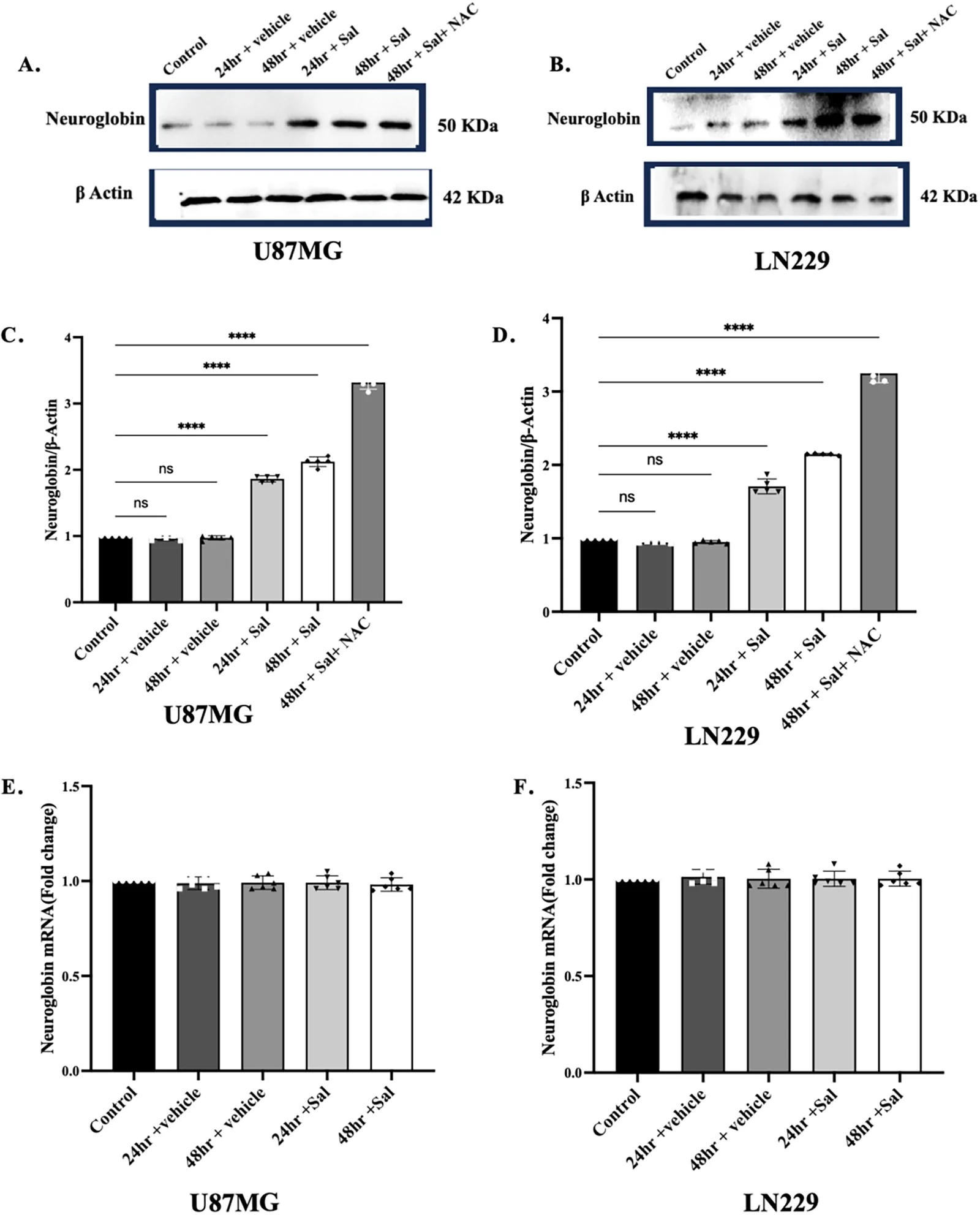
Salidroside increases Ngb expression in U87MG and LN229 cells **(A, B)** Relative protein expression of Ngb after salidroside treatment in U87MG and LN229 cells as detected by immunoblotting. β- actin was considered as loading control. **(C, D)** Quantitative protein fold change in Ngb expression in U87MG and LN229 cells. **(E, F)** Graph represents mRNA expression of Ngb quantified in U87MG and LN229 cells by real-time PCR. The data is presented as the fold change of transcripts for the target gene in the indicated groups normalized to GAPDH. ****P <0.0001 was considered significant when compared with control and study groups. ns, non-significant.

### 3.14 Salidroside increased the expression of cleaved caspase 3, cytochrome c in U87MG and LN229 cells

One of the most important features of potential anti-tumor drugs is fostering cell apoptosis. Since, salidroside supplementation triggered apoptosis in U87MG and LN229 cells as observed by Annexin V/PI staining, we examined the protein expression levels of cytochrome c and caspase-3, both of which are hallmark of apoptosis. The release of cytochrome c from mitochondria is a distinguishing feature of apoptosis. Cells treated with 80µM of salidroside for 24 and 48 h displayed significant increases in the expression levels of cleaved caspase-3 and cytochrome c as shown in **Figures 12A, B** indicating that salidroside promotes apoptosis of glioma cells. There is increase in cleaved caspase-3 and cytochrome c post supplementation with NAC. This effect is reversed on supplementation with CYP2E1 inhibitor. Inhibition of CYP2E1 attenuated the salidroside-associated changes in these apoptotic markers, providing additional pharmacological evidence supporting the involvement of CYP2E1 in the observed apoptotic response.

**Figure 12:**
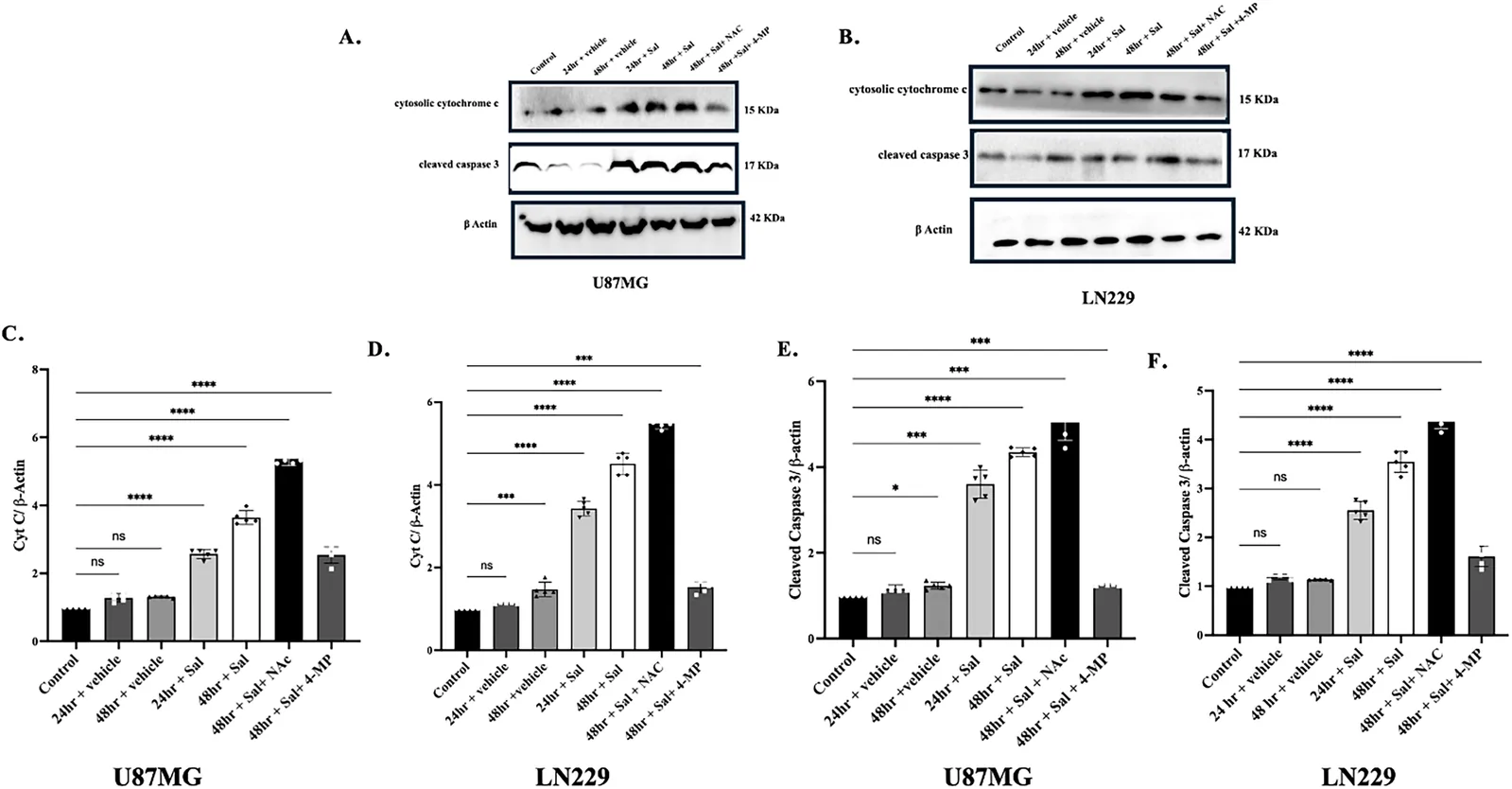
Cytochrome recycling could contribute to apoptosis in salidroside treated U87MG and LN229 cells **(A, B)** Representative images of western blot depicting cytosolic cytochrome c, cleaved caspase 3, and b- actin expression levels in U87MG and LN229 cells with and without salidroside (80μM) treatment. **(C, D)** Quantitative protein fold change cytochrome c in U87MG and LN229 cells **(E, F)** Cleaved caspase 3 protein expression in U87MG and LN229 cells. Graph represents mean ± SD. ****P <0.0001 ,***P<0.001 ,*P <0.05 was considered significant when compared with control and study groups. ns, non-significant. Sal, Salidroside; NAC, N-acetyl cysteine; 4-MP, 4-methylpyrazole.

## 4 Discussion

Salidroside has been extensively investigated for its neuroprotective, antioxidant, anti-inflammatory (6, 49–51), and anti-cancer properties across multiple preclinical models (52–58). Further, our previous studies on rodents demonstrated experimental evidence for the effective BBB permeability of salidroside, however, further brain distribution studies are warranted to establish glioma-specific CNS delivery (6). Its reported ability to preserve neuronal viability at pharmacologically relevant concentrations distinguishes it from many conventional chemotherapeutic agents and underscores its potential relevance in CNS malignancies. The computationally derived differential cytotoxicity suggesting preferential targeting of glioma cells while relatively sparing non-malignant neural cells, represent a promising preliminary observation of the present investigation. While these computational findings provide a preliminary mechanistic basis, it is imperative to acknowledge that such predictions necessitate systemic experimental validation to establish biological relevance and translational applicability.

Consistent with prior reports, the present study demonstrates a dose- and time-dependent reduction in viability of U87MG and LN229 glioma cells following salidroside exposure. However, the observed plateau in cytotoxicity beyond 80 µM, with viability remaining above LD_50_, suggests that salidroside may have limited efficacy as a monotherapy at higher concentrations. This ceiling effect highlights an important translational consideration and supports the rationale for evaluating salidroside in combination regimens. Indeed, emerging evidence from other cancer models indicates that salidroside can potentiate the efficacy of established chemotherapeutic agents, warranting similar combinatorial investigations in glioblastoma systems. Beyond its effects on proliferation, salidroside significantly reduced the migratory and invasive capacities of glioma cells at defined concentrations, suggesting a potential role in modulating tumor progression rather than solely inducing cytotoxicity. These findings may be particularly relevant given the highly invasive nature of glioblastoma. Which contributes to poor clinical outcomes.

A notable aspect of salidroside is its context-dependent activity under hypoxic conditions. While prior studies have demonstrated cytoprotective effects in neuronal models of hypoxia and ischemia (59), the present findings suggest that salidroside may exert opposing effects in glioma cells. This apparent dichotomy may reflect differences in cellular metabolic states and downstream signaling pathways, although the precise mechanisms remain to be clarified. In this context, the present study explored the involvement of CYP2E1, a cytochrome P450 isoenzyme implicated in oxidative metabolism and redox homeostasis. The observed increase in CYP2E1 activity following salidroside treatment, accompanied by NADPH consumption, suggests that salidroside may influence intracellular redox balance that was seen in the DCFDA assay that the ROS levels were increased post supplementation with salidroside for 48h. Given the central role of NAD(P)H-dependent systems in maintaining cellular oxidative status (60), these findings are consistent with a potential shift toward a pro-oxidant intracellular environment. However, whether CYP2E1 activation is a direct effect of salidroside or a secondary cellular response remains to be determined.

Complementing the experimental findings, in-silico analyses indicated a preferential interaction between salidroside and the reduced (Fe²^+^) form of Ngb. The predicted binding within the heme pocket, including potential interactions with key residues such as His64, suggests a possible influence on Ngb redox behavior. While these computational observations provide a mechanistic hypothesis, it is important to emphasize that docking results do not establish functional modulation and require biochemical validation. Ngb has been implicated in cellular oxygen homeostasis and redox signaling (61), with emerging evidence suggesting altered expression in glioma (11). Ngb facilitates the release of cytochrome C from mitochondria by triggering the intrinsic apoptotic pathway. Furthermore, caspase-8 is first activated in the extrinsic apoptotic pathway, which sets off a series of downstream signaling processes culminating in apoptosis. Consistent with this, the present study observed a grade-dependent decrease in Ngb expression and an increase following salidroside treatment. The absence of corresponding changes at the mRNA level suggests post-translational regulation, although the underlying mechanisms remain unclear. It is therefore premature to conclude a direct causal link between Ngb modulation and salidroside-induced cytotoxicity, but the association warrants further investigation. Markers of oxidative stress and apoptosis including increased lipid peroxidation (62, 63), altered MMP, elevated cytosolic cytochrome c, and caspase-3 activation, were observed following salidroside exposure while the effect was reversed when the cells were supplemented with salidroside in the presence of CYP2E1 inhibitor 4-MP. Together, these findings are consistent with activation of mitochondrial apoptotic pathways. However, the extent to which these effects are mediated CYP2E1-neuroglobin-cytochrome c pathway as a mechanistically supported association rather than a conclusively established linear causal pathway.

From a translational perspective, the predicted pharmacokinetic properties of salidroside are encouraging. Its aqueous solubility, lack of predicted cytochrome P450 inhibition, and potential for BBB penetration suggests a favourable drug-like profile. Nonetheless, these predictions require rigorous in vivo validation, particularly given the complexity of CNS drug delivery and the heterogeneity of the blood-tumor barrier in GB. Importantly, the predicted low cytotoxicity in non-malignant cell lines supports the possibility of a therapeutic window, although the absence of CNS-specific cell types in these analyses limits definitive conclusions regarding neurotoxicity. Future studies incorporating patient-derived primary cell culture and organotypic models will be essential to better define safety profiles. Taken together, the present findings suggest that salidroside exerts multi-faceted effects on glioma cells, potentially involving modulation of redox homeostasis, mitochondrial function, and invasive behaviour. Rather than acting through a single dominant pathway, salidroside may function as a pleiotropic modulator of cellular stress responses. This raises the possibility that its biological effects are dose- and context-dependent, consistent with a hormetic model in which low doses promote cytoprotection while higher doses induce oxidative stress and cell death.

Despite significant findings, the study has several limitations. Most importantly, the findings of this study are based on *in vitro* experiments and computational analyses, and therefore, their translational relevance remains to be established. Other limitations that need to be addressed are absence of animal validation, lack of pharmacokinetic measurements, absence of combination therapy studies and validation in patient-derived primary culture/organoid models. Further investigations, including *in vivo* studies and detailed mechanistic analyses, will be necessary to validate these observations and to establish the therapeutic potential of salidroside in GB.

Thus future investigations should focus on: (i) validating the role of CYP2E1 and Ngb in mediating salidroside responses, (ii) defining combinatorial therapeutic strategies, and (iii) evaluating pharmacokinetics and efficacy in in vivo GB models (iv) As such, the computationally predicted BBB permeability of salidroside should be interpreted as a preliminary indicator warranting further experimental validation through in vivo pharmacokinetic and biodistribution studies, rather than as confirmation of effective drug delivery. Such studies will be critical to determine whether the promising preclinical profile of salidroside can be translated into clinically meaningful therapeutic applications.

## 5 Conclusion

Taken together, the present study demonstrates that salidroside is associated with reduced cell viability, migration, and invasion in U87MG and LN229 human glioma cells, accompanied by the activation of apoptotic pathways (**Figure 13**). These effects were observed alongside increased CYP2E1 activity, alterations in intracellular NADPH levels, and changes in cellular redox status, suggesting a potential link between salidroside exposure and oxidative stress-mediated cellular responses. Salidroside treatment was further associated with mitochondrial dysfunction, as indicated by reduced mitochondrial membrane potential, increased lipid peroxidation, and elevated cytosolic cytochrome c levels, which are consistent with the activation of intrinsic apoptotic signaling. In addition, in-silico analyses suggest that salidroside may interact preferentially with the reduced form of Ngb, raising the possibility that Ngb-mediated redox processes could be influenced under these conditions. However, these computational findings require experimental validation to establish their functional relevance. Collectively, these observations suggest that salidroside may exert multi-faceted effects on glioma cells, potentially involving modulation of redox homeostasis, mitochondrial function, and apoptotic signaling pathways. Rather than indicating a single dominant mechanism, the data support a model in which multiple interconnected processes contribute to the observed cellular responses. 

**Figure 13:**
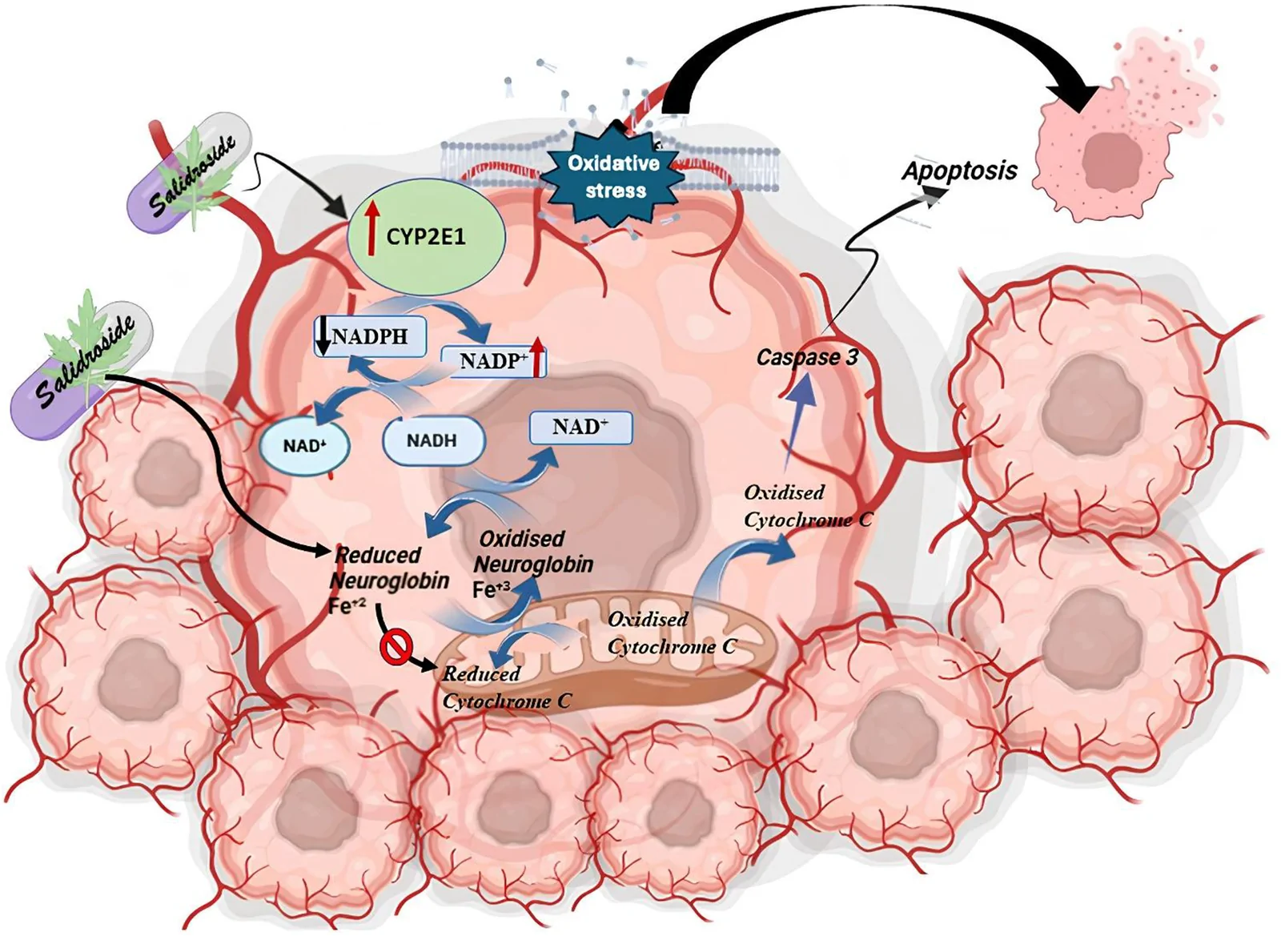
Schematic diagram representing the mechanism of action of salidroside in glioma cells.

## Data Availability

The original contributions presented in the study are included in the article/**Supplementary Material**, further inquiries can be directed to the corresponding author.
