# Correction: Salidroside inhibits glioma cell migration and proliferation through CYP2E1-neuroglobin-cytochrome c mediated redox axis

**DOI:** 10.3389/fonc.2026.2021223

**Published:** 2026-10-22

**Authors:** Lakshmi Priya Panda, Kalpana Barhwal, Amit Kumar, Suryanarayan Biswal, Dev Kumar Tripathy, Mukund Namdev Sable, Rabi Narayan Sahu, Gaurav Chhabra

**Affiliations:** 1 Department of Physiology, All India Institute of Medical Sciences, Bhubaneswar, India; 2 Department of Human Genetics and Molecular Medicine, Central University of Punjab, Bhatinda, India; 3 Department of Pathology, All India Institute of Medical Sciences, Bhubaneswar, India; 4 Department of Neurosurgery, All India Institute of Medical Sciences, Bhubaneswar, India

**Keywords:** glioblastoma, neuroglobin, oxidative stress, redox imbalance, salidroside

There was a mistake in **Figure 12** as published. The **Figure 12** displays raw blots which were uploaded as supplementary images.

**Figure 12:**
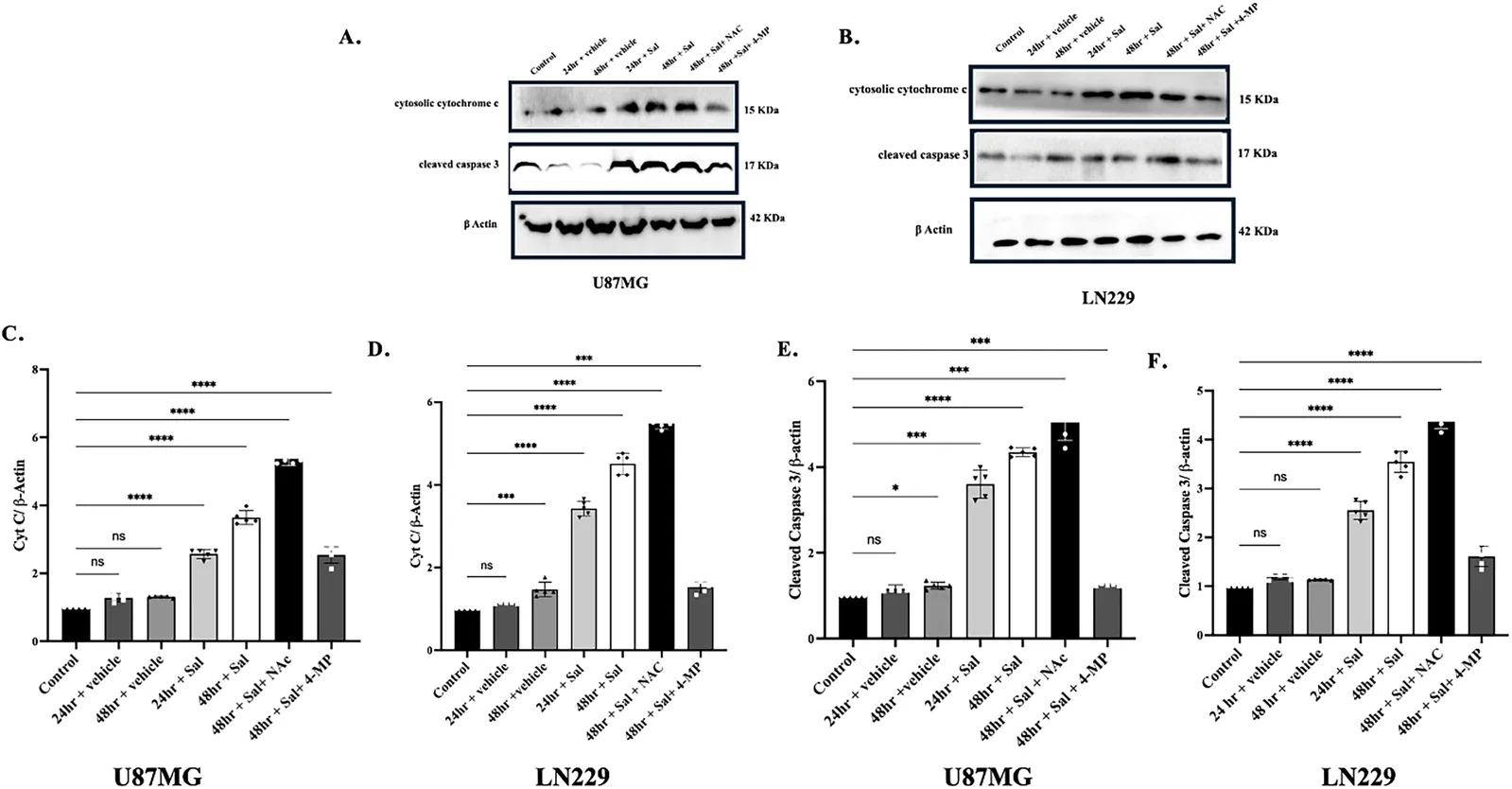
Cytochrome recycling could contribute to apoptosis in salidroside treated U87MG and LN229 cells **(A, B)** Representative images of western blot depicting cytosolic cytochrome c, cleaved caspase 3, and β- actin expression levels in U87MG and LN229 cells with and without salidroside (80µM) treatment. **(C, D)** Quantitative protein fold change cytochrome c in U87MG and LN229 cells **(E, F)** Cleaved caspase 3 protein expression in U87MG and LN229 cells. Graph represents mean ± SD. ****P <0.0001 ,***P<0.001 ,*P <0.05 was considered significant when compared with control and study groups. ns, non-significant. Sal, Salidroside; NAC, N-acetyl cysteine; 4-MP, 4-methylpyrazole.

The corrected **Figure 12** appears below.

The original version of this article has been updated

