# Supplementary material for: Salidroside inhibits glioma cell migration and proliferation through CYP2E1-neuroglobin-cytochrome c mediated redox axis
Source: Front Oncol. 2026 Oct 1;16:1929709. doi: 10.3389/fonc.2026.1929709 (PMC13668574; doi:10.3389/fonc.2026.1929709)
Supplement: Supplementary file 1 [file DataSheet1.docx]

**Supplementary Figures and Tables**

| Parameter | Fe²⁺ Ngb — Reduced | Fe³⁺ Ngb — Oxidized |
| --- | --- | --- |
| Total docked poses generated | 310 | 165 |
| Best binding free energy (kcal/mol) | −7.04 | −6.82 |
| Final intermolecular energy (kcal/mol) | −10.02 | −9.81 |
| vdW + H-bond + desolvation (kcal/mol) | −9.36 | −9.20 |
| Electrostatic energy (kcal/mol) | −0.66 | −0.61 |
| Top 4 pose energies (kcal/mol) | −7.04, −6.92, −6.90, −6.88 (Δ=0.16) | −6.82, −6.74, −6.64, −6.60 (Δ=0.22) |
| Pose energy distribution | Broad, dense cluster (−7 to −12) | Narrow, steep gradient |
| Binding landscape | Stable, conformationally consistent | Reduced flexibility |

**Table 1. Summary of docking parameters for Salidroside with Fe²⁺ and Fe³⁺ forms of Neuroglobin**

| **Interaction Feature** | **Fe²⁺ Ngb— Reduced** | **Fe³⁺ Ngb—Oxidized** |
| --- | --- | --- |
| Number of hydrogen bonds | 8 | 4 |
| H-bond distance range (Å) | 2.17–3.22 | 2.45–3.10 |
| Shortest H-bond (Å) | 2.17 (strong, direct) | 2.45 |
| Key H-bond residues | Leu41(B), Val99(B), Gln43(B) ×3, Lys95(B), His64(B) | Arg10(A), Ser17(A), Thr77(A) ×2 |
| Hydrophobic contacts | Phe42(B), Lys95(B), mHem152(B) | Trp13(A), Arg14(A), Asp73(A), Val76(A) |
| Direct Fe coordination | Yes — Fe–His64–Ligand at 2.17 Å | Absent |
| Binding pocket location | Deep heme-binding cleft | Outer polar surface |
| Overall binding strength | Strong | Moderate |

**Table 2. Comparative interaction analysis of Salidroside with Fe²⁺ and Fe³⁺ Neuroglobin (LigPlot⁺)**

| **Property** | **Value / Classification** |
| --- | --- |
| **Molecular weight (g/mol)** | **300.30** |
| **TPSA (Å²)** | **119.61** |
| **H-bond donors / acceptors** | **5 / 7** |
| **Rotatable bonds** | **5** |
| **Fraction Csp³** | **0.57** |
| **Consensus log S (ESOL)** | **−0.12 — Very Soluble** |
| **Log S SILICOS-IT** | **−0.44 — Soluble** |
| **GI absorption** | **High** |
| **Plasma protein binding** | **Low** |
| **P-gp substrate** | **Yes** |
| **Log Kp — skin permeability (cm/s)** | **−8.68** |
| **CYP450 inhibition (1A2, 2C9, 2C19, 2D6, 3A4)** | **None** |
| **Primary metabolic pathway** | **Phase II — UGT glucuronidation** |
| **Primary elimination** | **Renal excretion** |
| ***Bioavailability score*** | ***0.55*** |
| **Synthetic accessibility score** | **4.25 — Moderate** |
| **Drug-likeness rules** | **Lipinski ✓ Veber ✓ Egan ✓ Muegge ✓ (minor Ghose violation)** |
| **PAINS / Brenk alerts** | **None — clean scaffold** |
| **Classification** | **Lead-like** |

**Table 3. Physicochemical and drug-likeness properties of Salidroside (SwissADME / admetSAR 2.0)**

**Figure 1. Drug-likeness and physicochemical profile of Salidroside** (admetSAR 2.0 and SwissADME . Left: BOILED-Egg radar plot showing drug-likeness across lipophilicity (LIPO), water solubility (INSOLU), molecular size, polarity (POLAR), flexibility (FLEX), and saturation (INSATU). Right: tabulated physicochemical, pharmacokinetic, drug-likeness, and medicinal chemistry properties.

**A.**

**B.**

**C.**

**Figure 2. Pharmacokinetic, toxicological, and BBB permeability profile of Salidroside.** (A) pkCSM -predicted ADMET parameters. (B) Toxicological assessment by ProTox-3 and pkCSM , showing predicted LD₅₀ > 5000 mg/kg (Toxicity Class 5), absence of hepatotoxicity, cytotoxicity, carcinogenicity, hERG inhibition, and skin sensitization. (C) B3DB -predicted BBB permeability: log BB = −0.76689, classified as BBB Permeable.

| **Pa** | **Pi** | **Cell-line** | **Description** | **Tissue/Organ** | **Type** | **IAP*** |
| --- | --- | --- | --- | --- | --- | --- |
| 0.322 | 0.007 | NHDF | Fibroblast | Skin | Normal | 0.953 |
| 0.206 | 0.004 | RWPE-1 | Prostatic epithelial cell line | Prostate | Normal | 0.922 |
| 0.468 | 0.031 | ATH-8 | HTLV-I-infected human T-cell line | Peripheral blood | Normal | 0.964 |
| 0.254 | 0.221 | RD | Rhabdomyosarcoma | Muscle | Normal | 0.828 |
| 0.008 | 0.004 | CuFi-1 | Cystic Fibrosis | Lung; Bronchus; Epithelium | Normal | 1 |
| 0.707 | 0.005 | MRC5 | Embryonic lung fibroblast | Lung | Normal | 0.916 |
| 0.564 | 0.005 | HEL299 | Fibroblasts | Lung | Normal | 0.899 |
| 0.171 | 0.133 | IMR-90 | Embryonic lung fibroblast | Lung | Normal | 0.879 |
| 0.163 | 0.081 | FHC | Epithelial cell | Large intestine; Colon | Normal | 0.82 |
| 0.222 | 0.022 | HK-2 | Papilloma | Kidney | Normal | 0.879 |
| 0.016 | 0.002 | CNE2Z | Hybrid cell line | Hybrid | Normal | 1 |
| 0.07 | 0.021 | WIL2-NS | Lymphoblastoid cell | Haematopoietic, lymphoid tissue | Normal | 0.979 |
| 0.184 | 0.022 | BJ | Foreskin fibroblast | Foreskin | Normal | 0.955 |
| 0.134 | 0.058 | 4T1 | Epithelial mammary gland cells | Breast | Normal | 0.887 |

**Table 4. Salidroside shows non-toxic or weak activity in normal cell lines, indicating excellent cytocompatibility (5A)** **Predicted cytotoxicity of Salidroside against normal cell lines (CLC-Pred).** Pa = Probability of Activity (0→1; closer to 1 indicates higher likelihood of activity); Pi = Probability of Inactivity (0→1; closer to 1 indicates higher likelihood of inactivity); IAP = Invariant Accuracy of Prediction (higher IAP reflects greater model reliability). All cell lines are of normal tissue origin. Predictions were generated using the CLC-Pred server based on the canonical SMILES of Salidroside.
